# Olive Oil as a Modulator of Gut Microbiota and Intestinal Health: A Narrative Review from Microbial Metabolism to Host Responses

**DOI:** 10.3390/nu18142235

**Published:** 2026-07-09

**Authors:** Luna Barrera-Chamorro, Teresa Gonzalez-de la Rosa, Jose L. del Rio-Vazquez, Maria Torrecillas-Lopez, Elvira Marquez-Paradas, Carmen M. Claro-Cala, Sergio Montserrat-de la Paz, Maria D. Navarro-Hortal

**Affiliations:** 1 Department of Medical Biochemistry, Molecular Biology, and Immunology, School of Medicine, University of Seville, 41009 Seville, Spain; lbarrera1@us.es (L.B.-C.); mgonzalez18@us.es (T.G.-d.l.R.); jdelrio3@us.es (J.L.d.R.-V.); mtorrecillas2@us.es (M.T.-L.); emarquez5@us.es (E.M.-P.); delapaz@us.es (S.M.-d.l.P.); 2 Instituto de Biomedicina de Sevilla (IBiS), Hospital Universitario Virgen del Rocio, CSIC, University of Seville, 41013 Seville, Spain; cmclaro@us.es; 3 Department of Pharmacology, Pediatrics, and Radiology, School of Medicine, University of Seville, 41009 Seville, Spain

**Keywords:** extra virgin olive oil, virgin olive oil, gut microbiota, intestinal barrier, olive phenolics, hydroxytyrosol, inflammatory bowel disease, short-chain fatty acids, gut–brain axis, Mediterranean diet

## Abstract

Olive oil, particularly virgin (VOO) and extra-virgin olive oil (EVOO), is a central component of the Mediterranean diet and has been associated with cardiometabolic, anti-inflammatory, and intestinal health benefits. Increasing evidence suggests that these effects may involve interactions with the gut microbiota, intestinal barrier, and host inflammatory pathways. This narrative review summarizes current evidence on the impact of olive oil, olive-derived phenolics, and olive oil-rich dietary patterns on gut microbiota modulation, barrier function, inflammatory bowel diseases, and related systemic outcomes. The available literature indicates that olive oil may interact with the gut ecosystem through both its oleic acid-rich lipid matrix and its minor phenolic fraction. VOO and EVOO appear more consistently associated than refined oils with microbial or microbial metabolite profiles related to saccharolytic metabolism, short-chain fatty acid production, mucus-layer dynamics, and anti-inflammatory intestinal environments. Olive-derived phenolics, including hydroxytyrosol, tyrosol, oleuropein derivatives, and oleocanthal, can undergo microbial biotransformation and may influence bile acid metabolism, epithelial barrier integrity, and inflammatory signaling. Whole EVOO evidence is strongest in experimental colitis models, whereas human evidence mainly supports effects on postprandial endotoxemia, lipid oxidation, and selected inflammatory markers. However, findings remain heterogeneous and depend on oil quality, phenolic composition, comparator fat, dietary context, and host condition. Well-controlled human studies directly comparing EVOO, VOO, refined olive oil, and oleic acid-rich controls are needed to clarify reproducible microbiota-mediated effects and their relevance to intestinal and systemic health.

## 1. Introduction

Diet is a major determinant of human health and one of the most relevant modifiable factors influencing the risk of chronic non-communicable diseases. Among different dietary patterns, the Mediterranean diet (MD) has consistently been associated with reduced incidence of cardiovascular disease, metabolic disorders, neurodegenerative diseases, and overall mortality. A defining characteristic of the MD is the high consumption of olive oil, particularly extra virgin olive oil (EVOO), which represents the main source of dietary fat and is considered a key contributor to the health benefits of this dietary pattern [1,2]. This evidence is supported by major dietary intervention studies such as “PREvención con DIeta MEDiterránea” (PREDIMED), in which an MD supplemented with EVOO reduced major cardiovascular events in individuals at high cardiovascular risk [1], and “Coronary Diet Intervention With Olive Oil and Cardiovascular Prevention” (CORDIOPREV), which evaluated a Mediterranean diet rich in olive oil for secondary cardiovascular prevention in patients with established coronary heart disease [3]. In line with these intervention data, prospective cohort studies have associated higher olive oil consumption with lower all-cause, cardiovascular, and cancer mortality in Mediterranean populations [4], with similar inverse associations reported in large non-Mediterranean cohorts [5]. The beneficial effects of olive oil are generally attributed to the combination of an oleic acid-rich lipid fraction and a minor bioactive fraction that includes phenolic compounds, tocopherols, phytosterols, and other bioactive molecules. Among these, EVOO phenolics have received particular attention because of their antioxidant, anti-inflammatory, and immunomodulatory properties, and may act synergistically with the lipid fraction [6,7,8].

In recent years, gut microbiota has emerged as a central mediator linking diet and host health. The intestinal microbiota plays a crucial role in nutrient metabolism, maintenance of intestinal barrier integrity, immune system regulation, and control of systemic inflammatory responses [9,10]. Alterations in gut microbiota composition and function have been associated with a wide range of pathological conditions, including inflammatory bowel diseases, obesity, metabolic syndrome, and neurological disorders [11]. Diet is one of the most powerful modulators of gut microbiota composition and activity. Both the amount and the quality of dietary fat have been shown to influence microbial diversity and the abundance of specific bacterial taxa [12]. In this context, increasing evidence suggests that olive oil exerts specific effects on the gut microbiota that differ from those induced by other dietary fat sources, particularly saturated fats. These effects appear to be mediated not only by oleic acid but also by olive oil phenolic compounds, which can reach the colon and interact with gut microorganisms or host cells [13,14]. Based on the abovementioned, this narrative review aims to summarize and critically discuss current evidence on the impact of olive oil and olive-derived compounds on gut microbiota modulation, intestinal barrier function, inflammatory bowel diseases, and related systemic outcomes.

## 2. Methodology

A literature search was performed using combinations of terms related to olive oil and its major constituents, including “olive oil”, “extra virgin olive oil”, “virgin olive oil”, “EVOO”, “*Olea europaea*”, “olive oil polyphenols”, “hydroxytyrosol”, “oleuropein”, “tyrosol”, “oleocanthal”, “oleic acid” and “monounsaturated fatty acids”, combined with terms related to gut and inflammatory outcomes, such as “gut microbiota”, “gut microbiome”, “fecal microbiota”, “intestinal barrier”, “intestinal permeability”, “tight junction”, “lipopolysaccharide”, “LPS”, “inflammatory bowel disease”, “ulcerative colitis”, “Crohn disease”, “colitis”, “dysbiosis” and “intestinal inflammation”. The search was conducted in PubMed, Scopus, and Web of Science and included studies published up to May 2026. Duplicate records retrieved from different databases were identified and removed prior to the screening process. Studies were included if they investigated olive oil, olive-derived phenolics, or olive oil-based dietary patterns in relation to gut microbiota composition, microbial metabolites, intestinal barrier function, or related health outcomes. Reviews, editorials, and non-English publications were excluded. The evidence was interpreted according to study design and biological relevance, including human intervention and observational studies, preclinical dietary fat and disease models, and in vitro or ex vivo mechanistic studies. Preclinical and mechanistic evidence was considered particularly relevant when it provided information on microbiota modulation, phenolic biotransformation, intestinal barrier integrity, or inflammatory pathways.

## 3. Olive Oil: Definition, Composition, and General Health Benefits

### 3.1. Definition

According to the definition provided by the International Olive Council, VOOs are “the oils obtained from the fruit of the olive tree (*Olea europaea* L.) solely by mechanical or other physical means under conditions, particularly thermal conditions, that do not lead to alterations in the oil, and which have not undergone any treatment other than washing, decantation, centrifugation and filtration”. Virgin olive oils are classified as extra EVOOs or VOOs according to both sensory evaluation and physicochemical quality parameters. EVOOs must have a free acidity of ≤0.8%, whereas VOOs may have a free acidity of up to 2.0% [15].

### 3.2. Composition and General Health Benefits

As mentioned, olive oil is a central component of the MD and one of the most extensively studied dietary fats due to its well-established health benefits. Its biological effects are largely determined by a complex chemical composition that includes a major lipid fraction and a minor non-lipid fraction composed of a wide variety of bioactive compounds. The qualitative and quantitative composition of olive oil depends on multiple factors, including olive cultivar, degree of ripeness, agronomic practices, climatic conditions, and processing methods, with EVOOs being the least processed and richest in bioactive compounds [16].

The lipid fraction accounts for approximately 98–99% of olive oil and consists mainly of triglycerides, with fatty acids esterified to a glycerol backbone. The predominant fatty acid in olive oil is oleic acid (C18:1 *n*-9), a monounsaturated fatty acid (MUFA) that typically represents 55–83% of total fatty acids, depending on the variety and geographical origin [16,17]. This high oleic acid content distinguishes olive oil from other dietary fats and is considered a major contributor to its cardiometabolic benefits. In addition to oleic acid, olive oil contains smaller amounts of other fatty acids, including palmitic acid (C16:0, 7–20%), stearic acid (C18:0, 0.5–5%), linoleic acid (C18:2 *n*-6, 3–21%), and α-linolenic acid (C18:3 *n*-3, <1%) [17,18,19], being the last one a potent fatty acid on reduction in LDL-cholesterol (LDL-c) [18]. The relatively low proportion of saturated fatty acids, together with a favorable MUFA-to-saturated fatty acid ratio and a high ω6/ω3 ratio, has been associated with improved lipid profiles, enhanced insulin sensitivity, and protective effects against coronary, autoimmune, and inflammatory disorders, as well as anti-thrombotic properties and regulation of blood pressure, compared to diets rich in saturated fats [5,16]. Beyond classical cardiometabolic endpoints, VOO has also been linked to systemic mechanisms relevant to healthy aging, including oxidative stress, mitochondrial function, inflammatory regulation, tissue preservation, and survival-related outcomes [20,21]. Lifelong-feeding studies in rats suggest that diets based on virgin olive oil may reduce age-specific mortality compared with sunflower oil-based diets [22].

Although the non-lipid fraction of olive oil represents only 1–2% of its total weight, it contains a wide range of biologically active compounds that play a key role in its health-promoting properties. This fraction includes phenolic compounds, tocopherols, phytosterols, pigments, squalene, and other minor constituents [16,23]. Phenolic compounds are among the most studied components of olive oil, particularly in EVOO. They include simple phenols (hydroxytyrosol (HT) and tyrosol), secoiridoids (oleuropein and ligstroside derivatives), phenolic acids, flavonoids, and lignans [24]. The total phenolic content of EVOO can vary widely, typically ranging from 30 to over 800 mg/kg, depending on production and storage conditions [23,25,26]. The European Food Safety Authority (EFSA) has recognized the health relevance of olive oil phenolics, authorizing a health claim stating that the daily consumption of olive oil containing at least 5 mg of HT and its derivatives contributes to the protection of blood lipids from oxidative stress [25]. Olive oil is also an important dietary source of tocopherols, particularly α-tocopherol (vitamin E), which contributes to antioxidant protection and lipid stability. EVOO typically contains 100–300 mg/kg of α-tocopherol, depending on cultivar and processing [27,28]. Other minor components include phytosterols, which may contribute to cholesterol-lowering effects, and squalene, a triterpene abundant in olive oil that has been associated with antioxidants and potential chemoprotective properties. Pigments such as chlorophylls and carotenoids, while present in small amounts, also contribute to antioxidant activity and oil stability [16]. Table 1 summarizes the main physicochemical characteristics, fatty acid composition, and minor bioactive constituents of virgin olive oil (VOO) and extra virgin olive oil (EVOO), highlighting the main quality and compositional features that distinguish both categories.

### 3.3. Nutritional Value and Dietary Intake

From a nutritional perspective, olive oil is an energy-dense food, providing approximately 9 kcal/g, but its health effects are determined primarily by fat quality rather than total fat content. In Mediterranean populations, olive oil intake typically ranges from 25 to 50 mL per day, corresponding to approximately 2–4 tablespoons, and may exceed this amount in traditional dietary patterns [1,32]. Based on the reported phenolic content of EVOO (100–800 mg/kg), a daily intake of 25–50 mL (23–46 g of oil) would provide an estimated phenolic intake ranging from approximately 2.3 to 36.8 mg/day, depending on cultivar, agronomic conditions, extraction technology, and storage. This highlights the relatively low physiological exposure to olive oil phenolics through a typical Mediterranean diet compared with the substantially higher doses commonly employed in preclinical and in vitro studies [16,23]. Nonetheless, the level of intake is nutritionally relevant because it can represent a substantial proportion of total dietary fat while replacing less favorable lipid sources such as butter, lard, or other saturated fat-rich fats. Therefore, olive oil should be considered not simply as an added source of energy, but as a dietary fat whose impact depends on the food matrix it replaces.

Beyond its caloric contribution, olive oil provides a characteristic nutrient profile dominated by oleic acid, together with smaller amounts of essential polyunsaturated fatty acids and minor bioactive compounds such as tocopherols, phytosterols, squalene, and phenolics, particularly in VOO and EVOO [16,23]. This composition makes olive oil nutritionally distinct from refined seed oils, animal fats, or tropical oils, even when total fat intake is similar. Its use as the main culinary fat in MDs is also relevant because it is commonly consumed with vegetables, legumes, whole grains, and other plant foods, contributing to palatability, dietary adherence, and the broader dietary context in which microbiota-related effects are observed [1,13].

Clinical and epidemiological studies consistently indicate that replacing saturated fats with olive oil, particularly EVOO, improves cardiometabolic risk markers without promoting weight gain when consumed within an overall balanced diet [1,5,8]. Moreover, VOO intake has been associated with higher gut microbial diversity, while Mediterranean dietary adherence has been linked to short-chain fatty acids (SCFAs)-producing and saccharolytic microbial profiles, supporting its role as a functional dietary fat rather than a neutral energy source [13,33]. However, these associations should be interpreted in relation to oil quality and dietary context: EVOO provides phenolic compounds, tocopherols, and other minor constituents that are largely reduced during refining, whereas the effects of olive oil consumed within an MD cannot be fully separated from fiber, plant polyphenols, and other microbiota-active components of the diet.

### 3.4. Digestion, Bioaccessibility, and Bioavailability of Olive Oil Components

Following ingestion, the lipid fraction of VOO and EVOO is efficiently digested in the gastrointestinal tract. Triacylglycerols are hydrolyzed by gastric and pancreatic lipases into free fatty acids and 2-monoacylglycerols, which are incorporated into mixed micelles and absorbed by enterocytes. Experimental in vitro digestion studies have shown that olive oil undergoes extensive lipolysis, resulting in high bioaccessibility of its main lipid components. Moreover, lipid bioaccessibility is greater than that reported for more highly unsaturated edible oils and is positively associated with the proportion of oleic acid, while enrichment with antioxidant phenolic compounds does not impair lipolysis or lipid bioaccessibility [34].

The gastrointestinal fate of olive oil phenolics is more complex. Using the standardized INFOGEST digestion model, Reboredo-Rodríguez et al. demonstrated that gastrointestinal digestion induces extensive hydrolysis of secoiridoids, generating simpler phenols such as HT and tyrosol. They also showed that bioaccessibility differs markedly among phenolic families: simple phenols and phenolic acids were mainly recovered in the aqueous fraction after digestion, whereas lignans remained comparatively stable and associated with the oily fraction. These findings indicate that the initial phenolic profile of EVOO strongly influences the fraction of compounds potentially available for intestinal absorption [35]. Human intervention studies further support the intestinal absorption of olive oil phenolics. Vissers et al. estimated that approximately 55–66% of ingested olive oil phenols are absorbed in the small intestine, with extensive metabolic transformation occurring after absorption [36]. Consistently, intervention studies have shown that consumption of VOO increases circulating phenolic metabolites, mainly sulfated and glucuronidated derivatives of HT and tyrosol, while higher phenolic intake further increases their plasma concentrations, although considerable interindividual variability exists [37,38].

**Figure 1:**
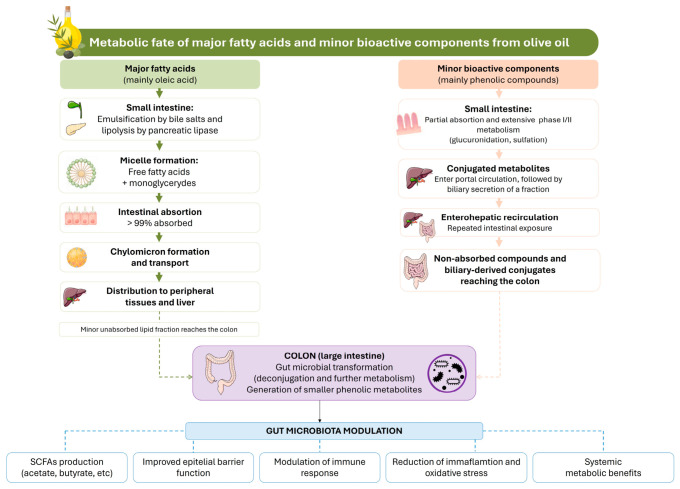
Metabolic fate of major fatty acids and minor bioactive components from olive oil.

Overall, the biological effects of VOO and EVOO not only depend on their chemical composition but also on the digestive stability, bioaccessibility, and bioavailability of their bioactive constituents. Importantly, because not all phenolic compounds are absorbed in the upper gastrointestinal tract, a proportion reaches the colon, where it becomes available for microbial metabolism, providing the basis for the microbiota-mediated mechanisms discussed in the following section. The metabolic fate of the major fatty acids and minor bioactive components of olive oil, from gastrointestinal digestion and absorption to microbial biotransformation and their contribution to gut microbiota modulation, is summarized in Figure 1.

## 4. Gut Microbiota and Its Role in Health

The gut microbiota comprises a complex and dynamic community of microorganisms, predominantly bacteria, but also archaea, viruses, and fungi, that inhabit the gastrointestinal tract and play a fundamental role in human health. The establishment of the intestinal microbiota is shaped by a wide range of factors, such as host genetics, age, environment, diet, and exposure to medications, particularly antibiotics [39]. In healthy adults, the gut microbiota is primarily dominated by the phyla Firmicutes and Bacteroidetes, followed by Actinobacteria, Proteobacteria, and Verrucomicrobia, although substantial interindividual variability exists in microbial composition and diversity [40,41]. Among the nearly 50 bacterial phyla described to date, Firmicutes and Bacteroidetes together account for more than 90% of the gut bacterial population. Firmicutes include genera such as *Lactobacillus*, *Clostridium*, and *Faecalibacterium*, which are actively involved in the fermentation of complex carbohydrates and the production of SCFAs. Bacteroidetes, represented by genera such as *Bacteroides* and *Prevotella*, play a key role in dietary fiber degradation and immune system modulation. The relative abundance between these two dominant phyla, often expressed as the Firmicutes-to-Bacteroidetes (F/B) ratio, has been widely used as an indicator of intestinal homeostasis and metabolic health, although its interpretation remains context-dependent [39,42].

One of the primary mechanisms through which gut microbiota influences host physiology is the fermentation of non-digestible dietary components, leading to the production of bioactive metabolites. Among these, SCFAs, mainly acetate, propionate, and butyrate, are particularly relevant. These metabolites serve as energy substrates for colonocytes, reinforce epithelial barrier function, and exert systemic effects on glucose and lipid metabolism, inflammatory pathways, and immune responses [43]. The gut microbiota also plays a central role in the development and regulation of the immune system. Commensal microorganisms promote immune tolerance by inducing regulatory T cells, modulating antigen-presenting cell activity, and limiting excessive inflammatory responses, thereby contributing to immune homeostasis [9]. Conversely, dysbiosis has been consistently linked to chronic inflammatory and metabolic disorders, including inflammatory bowel diseases, obesity, type 2 diabetes, cardiovascular disease, and autoimmune conditions [9]. In addition to immune regulation, the gut microbiota influences host metabolic health by modulating energy harvest from the diet, bile acid metabolism, and signaling pathways involved in insulin sensitivity and lipid homeostasis [44,45]. Microbial-derived metabolites such as secondary bile acids, indole derivatives, and trimethylamine-related compounds act as signaling molecules that interact with host receptors, thereby contributing to systemic metabolic and inflammatory regulation [46]. The integrity of the intestinal barrier represents another critical component of microbiota–host interactions. A healthy gut microbiota supports the maintenance of tight junction proteins, mucus layer integrity, and antimicrobial peptide production, limiting bacterial translocation and metabolic endotoxemia [47]. Disruption of this barrier has been linked to low-grade systemic inflammation and the pathogenesis of several cardiometabolic and inflammatory diseases [48]. Finally, growing evidence highlights the role of the gut microbiota in bidirectional communication with distant organs, including the brain, liver, and immune system, through neural, endocrine, and immune pathways. This gut–organ crosstalk further underscores the microbiota as a central regulator of human health and disease [11].

## 5. Olive Oil and Modulation of Gut Microbiota

The evidence linking olive oil to gut microbiota modulation is best interpreted by separating the matrices and experimental contexts in which it has been studied. Whole EVOO or VOO, refined olive oil (ROO), olive oil-rich Mediterranean dietary patterns, isolated phenolic compounds, olive leaf extracts, olive pomace, and other olive-derived by-products do not represent equivalent exposures. This distinction is important because microbial responses may reflect the oleic acid-rich lipid matrix, the phenolic and minor-compound fraction, the surrounding diet, or the physiological condition of the host.

For this reason, the following sections organize the evidence according to oil category, comparator fat, host context, phenolic biotransformation, and Mediterranean dietary pattern. This structure avoids treating olive oil as a single uniform intervention and allows microbiota findings to be interpreted together with microbial metabolites, barrier-related endpoints, and inflammatory outcomes.

### 5.1. Does the Type of Olive Oil Matter? Evidence from Extra Virgin, Virgin, and Refined Olive Oils

One of the most important issues in this field is whether all olive oils exert comparable effects on the gut microbiota. This question is especially relevant because EVOO, VOO, and ROOs share a broadly similar fatty acid profile but differ substantially in their content of phenolics and other minor compounds. Studies comparing these oils, therefore, provide a useful way to separate, at least partially, the contribution of the oleic acid-rich lipid fraction from that of the minor bioactive fraction.

The available comparative evidence indicates that the degree of oil refinement can influence the microbial response. In mice fed high-fat diets (HFDs) containing EVOO or ROO, fecal microbial profiles differed between the two olive oil matrices: EVOO produced a microbial configuration more clearly separated from the saturated fat reference pattern, whereas ROO showed a more intermediate response [49]. Although this study relied on culture-based methods and denaturing gradient gel electrophoresis (DGGE), which provide lower resolution than current high-throughput sequencing approaches, its value lies in the direct comparison between an unrefined and a refined olive oil matrix [49]. More detailed sequencing-based studies further support the distinction between EVOO and ROO [50,51]. In the comparison by Martínez et al., [50], ROO was associated with a less favorable microbial profile than EVOO, including some bacterial families showing positive associations with total cholesterol. Olid et al. [51] further extended this comparison in EVOO, ROO, and butter in a high-fat dietary setting. EVOO, ROO, and butter generated distinct fecal microbiota profiles at 6 weeks, with EVOO showing lower *Proteobacteria* than both butter and ROO. Differences between EVOO and ROO involved several taxa, including Rikenellaceae, Lactobacillaceae, Spiroplasmataceae, Enterobacteriaceae, *Alistipes*, *Lactobacillus*, *Rikenella*, *Desulfovibrio*, *Christensenella*, *Staphylococcus*, and *Desulfovibrio desulfuricans*. Because EVOO and ROO had broadly similar fatty acid profiles, these differences support the possibility that the minor compounds retained in EVOO contribute to microbiota modulation beyond the oleic acid-rich lipid matrix. However, these studies remain comparative and do not identify individual phenolics or other minor compounds as causal drivers.

A comparable distinction has also begun to emerge in human data. In the PREDIMED-Plus observational analysis, habitual intake of VOO/EVOO was evaluated separately from common olive oil, a category that includes more refined oils [33]. VOO/EVOO intake was associated with higher microbial diversity and more favorable cognitive trajectories, whereas common olive oil showed the opposite pattern, with lower alpha diversity and less favorable cognitive outcomes. The associated taxa also differed between categories. VOO/EVOO was linked with *Bacteroides*, *Phascolarctobacterium*, and *Acidaminococcus*, whereas common olive oil was associated with higher *Streptococcus* and lower *Faecalibacterium* [33]. Although these human associations cannot be directly mapped onto the animal studies, they point in a broadly consistent direction with the previous preclinical studies: less refined olive oil categories tend to associate with higher diversity and taxa related to microbial metabolic activity, whereas common or refined oils show associations with taxa more often discussed in relation to dysbiosis or reduced intestinal homeostasis. For example, lower *Faecalibacterium* may be relevant because this genus is commonly linked to butyrate production and anti-inflammatory intestinal environments [52], while higher *Streptococcus* is generally interpreted more cautiously as a potential marker of a less favorable microbial profile [33]. Likewise, the association of VOO/EVOO with *Phascolarctobacterium* may be relevant to succinate/propionate metabolism, although this should not be interpreted as proof of functional SCFA changes in the absence of metabolomic confirmation [53]. These taxonomic associations should nevertheless be interpreted with caution, since microbiota was assessed at baseline and the observational design does not allow longitudinal microbial changes or causal relationships to be established. Rather than defining a specific mechanism, these findings add human support to the idea that olive oil categories may differ in their associations with gut microbiota and should therefore be considered separately in future studies.

Beyond observational associations, the limited intervention evidence with whole EVOO provides a more direct, although still preliminary, view of the oil as actually consumed. These studies are less suited to separating the effects of individual fatty acids or phenolics, but they are more relevant for assessing whether EVOO intake can modify gut microbial or microbial metabolite outcomes in humans. In older patients with human immunodeficiency virus (HIV), 50 g/day EVOO for 12 weeks was associated with reduced total cholesterol, increased alpha diversity in men, and decreases in taxa interpreted as potentially pro-inflammatory, including Dethiosulfovibrionaceae, *Mogibacterium*, and *Coprococcus* [54]. Similarly, 30 mL/day polyphenol-rich EVOO for 100 days was associated with changes in both fecal and salivary microbiota, including increased fecal Bacteroidota and shifts in salivary Bacteroidota and Bacillota, together with reductions in HbA1c, LDL-c, and salivary interleukin (IL)-1β [55]. On the other hand, 40 mL/day EVOO for 14 days increased fecal total SCFA and the relative contribution of butyrate, while reducing the platelet-to-lymphocyte ratio as an inflammatory marker in patients with chronic kidney disease (CKD) undergoing hemodialysis [56]. Because bacterial composition, diversity, and taxonomic shifts were not assessed in this study, it should be interpreted as evidence for microbial metabolite modulation rather than direct microbiota profiling. Overall, these interventions add translational support to the idea that whole EVOO intake can influence selected microbial or microbial metabolite outcomes, but their uncontrolled or pre–post designs, small sample sizes, and specific clinical contexts prevent firm conclusions.

Taken together, the available preclinical and human data indicate that the microbial response to olive oil depends, at least in part, on oil quality and refinement status. It is summarized in Table 2. The strongest evidence supports a distinction between VOO/EVOO and more refined or common olive oils, while also showing that this distinction cannot yet be translated into a defined EVOO-specific microbial signature. Well-controlled human trials directly comparing EVOO with ROO, common olive oil, or oleic acid-rich control oils under matched dietary conditions are still needed.

### 5.2. Olive Oil Versus Other Dietary Fats: Fat Quality and High-Fat Diet Models

HFD models address a different question from studies focused on olive oil refinement. Here, the relevant issue is how olive oil behaves when it becomes part of an excessive-fat dietary environment and is compared with other lipid sources, including butter, lard, palm oil, coconut oil, soybean oil, perilla oil, safflower oil, fish oil, or broader Mediterranean-like fat blends. These models allow the effects of olive oil, EVOO, or VOO to be interpreted in relation to HFD-induced dysbiosis and host dysfunction, including altered microbial diversity, expansion of potentially pro-inflammatory taxa, metabolic stress, oxidative damage, endotoxemia, intestinal inflammation, and barrier-related impairment. They also introduce important variability related to the comparator fat, the type and quality of olive oil, the dose, the duration of feeding, and the host model. Therefore, the evidence should be read as a way to identify which features of HFD-induced dysbiosis or host dysfunction are attenuated, unchanged, or modified by olive oil.

Across comparisons with saturated fat-rich HFDs, olive oil generally produced microbial profiles distinct from those induced by butter, lard, or palm oil, often alongside less adverse metabolic or inflammatory readouts. In mice fed HFDs, butter produced the most marked disruption of fecal microbial profiles, whereas EVOO diverged more clearly from the butter-associated pattern, and ROO showed a less pronounced response [49]. A similar separation was observed when VOO was compared with butter: the saturated fat-rich diet showed a more unfavorable metabolic phenotype, including higher body weight, systolic blood pressure, and insulin, and this profile was accompanied by higher Desulfovibrionaceae/*Desulfovibrio* than in the VOO group [57]. As previously mentioned, this taxon is relevant because sulfate-reducing bacteria are often discussed in relation to endotoxin-associated metabolic inflammation. Other saturated fat comparisons support the same general direction. Olive oil generated a cecal microbial profile distinct from palm oil, with higher Bacteroidaceae/*Bacteroides* and lower adiposity, although diversity indices were not consistently higher in the olive oil group [58]. Similarly, HFDs based on olive oil, lard oil, or soybean oil produced different microbiota, lipid, and oxidative stress profiles, with the olive oil diet showing milder oxidative and metabolic disruption than lard oil for several outcomes, while still differing from the normal-fat control diet [59]. Thus, olive oil may shift the HFD response away from some adverse features associated with saturated fat-rich diets, but it does not fully normalize the microbial or metabolic profile.

The response of Bifidobacteria and Enterobacteriaceae illustrates this context dependency particularly well. At recommended intake, olive oil increased *Bifidobacterium* and improved insulin-related and hepatic metabolic readouts compared with coconut oil, whereas excessive intake of any tested oil reduced alpha diversity [60]. In contrast, when part of the lard in an HFD was replaced by perilla oil, olive oil, or safflower oil, olive oil reduced Enterobacteriaceae but did not increase Bifidobacteria, and perilla oil improved both microbial markers [61]. Bifidobacteria are generally associated with saccharolytic metabolism and intestinal homeostasis, whereas Enterobacteriaceae are facultative anaerobes that often expand under inflammatory or dysbiotic conditions. In the same study, olive oil also improved several HFD-induced alterations, including final body weight, liver weight, epididymal fat weight, serum leptin, colon length, macroscopic colon score, and serum endotoxin. However, the microbiota analysis was limited to the culture-based quantification of Bifidobacteria and Enterobacteriaceae, and the olive oil was commercially purchased and characterized mainly by its fatty acid profile, with oleic acid as the predominant fatty acid [61]. Therefore, olive oil can attenuate selected metabolic and intestinal alterations during HFD feeding, but it does not consistently produce the strongest bifidogenic or microbiota-associated response among dietary oils. The same caution applies to alpha diversity. In one HFD model, EVOO increased alpha diversity compared with high-fat lard and modified taxa such as *Bacteroides*, *Allobaculum*, *Lachnospiraceae*, and *Mucispirillum* [62]. In other models, olive oil modified specific taxa or host outcomes without consistently increasing diversity, as shown by the palm-oil comparison and the dose–response study described above [58,60]. These findings indicate that diversity is a context-dependent ecological marker, whereas the biological relevance of the response depends on which taxa change and whether those changes are accompanied by improvements in metabolic, inflammatory, or barrier-related outcomes.

Host readouts help clarify which microbial changes may be biologically meaningful. In the EVOO-versus-lard model, the EVOO-associated microbiota profile occurred together with increased colonic FoxP3 and IL-10, increased ileal RegIIIγ, and reduced hepatic LBP, linking the dietary fat source with mucosal immune regulation and lower exposure to gut-derived microbial products [62]. In female CD1 mice fed HFDs based on EVOO, coconut oil, or sunflower oil, EVOO did not prevent the HFD-induced reduction in richness/diversity, but it reduced Proteobacteria and potentially pro-inflammatory genera such as *Enterococcus*, *Staphylococcus*, and *Pseudomonas*, while better preserving *Akkermansia muciniphila* than coconut or sunflower oil [63]. Proteobacteria and facultative pathobiont genera are often interpreted as markers of dysbiosis or inflammatory pressure, whereas *Akkermansia muciniphila* is commonly linked to mucus-layer dynamics and metabolic health. These findings suggest that EVOO may influence microbial features related to inflammatory tone, barrier function, or metabolic endotoxemia even when global diversity is not preserved. Nevertheless, this does not mean that olive oil produces the strongest functional response in every setting: in Sprague–Dawley rats exposed to chronic mild stress, EVOO increased *Romboutsia*, *Akkermansia*, and *Ruminococcaceae_UCG_003*, but fish oil produced clearer improvements in depressive-like behavior [64]. Longer-term and host-vulnerable models further show that olive oil acts within a broader luminal environment. In BALB/c mice fed HFDs based on lard, olive oil, or soybean oil for 27 weeks, fecal microbiota, together with immunoglobulin (Ig) A coating and fecal long-chain fatty acids, were assessed. The high-olive oil diet generated microbiota patterns distinct from the high-lard and high-soybean oil diets, but the main finding was that fat source-dependent microbial shifts were more consistently associated with fecal long-chain fatty acid concentrations than with IgA coating [65]. This suggests that, under HFD conditions, the luminal lipid environment and unabsorbed fatty acids may contribute to microbiota restructuring alongside fatty acid composition itself. A useful example of this context-dependent microbial response comes from a *Muc2*^−/−^ mouse model fed isocaloric HFDs differing only in fat composition. In this setting, a Mediterranean-like fat blend, olive oil, corn oil, and milk fat produced distinct colonic microbiota profiles. The Mediterranean-like blend was associated with taxa such as *Lactobacillus animalis*, *Muribaculaceae*, and *Alistipes* spp., whereas the individual-fat diets, including olive oil, showed stronger associations with taxa such as Enterobacteriaceae and *Bacteroides massiliensis* [66]. In this mucus barrier-deficient model, the Enterobacteriaceae signal may indicate a more inflammation-prone luminal environment, while the association of the Mediterranean-like blend with *Lactobacillus* and Muribaculaceae is more compatible with mucosal adaptation and carbohydrate fermentation. Thus, from a microbiota perspective, this study suggests that olive oil enrichment alone does not necessarily reproduce the microbial configuration induced by a broader Mediterranean-like fatty acid profile [66].

Human dietary fat data provide a useful boundary for extrapolating these animal findings. Although olive oil was not specifically evaluated, a MUFA-rich diet in subjects at risk of metabolic syndrome did not reproduce the broader microbiota patterns reported in MD- or EVOO-related studies [67]. This supports an important distinction: effects observed with EVOO or olive oil-rich dietary patterns should not be reduced to MUFA content alone, because the whole-oil matrix, degree of refinement, phenolic fraction, and surrounding dietary pattern may all influence microbial response.

Viewed together, comparative fat studies suggest that olive oil can attenuate selected dysbiotic, inflammatory, or metabolic alterations during HFD feeding, particularly when compared with saturated fat-rich matrices. However, its effects remain conditional, depending on dose, oil matrix, comparator fat, host model, and analytical method, and they should not be reduced to either increased diversity or MUFA content. This distinction is essential because olive oil used as a fat source in experimental HFDs should not be assumed to reproduce the microbial effects of EVOO consumed within a Mediterranean dietary pattern. Preclinical studies comparing olive oil with other dietary fats are summarized in Table 3.

### 5.3. Host Context and Disease Models: Metabolic, Immune, and Inflammatory Settings

The response to olive oil is also shaped by the physiological condition of the host. Beyond HFD comparisons, several disease-specific models have tested EVOO or olive oil-derived components in settings of metabolic syndrome, diabetes, hypertension, neuroinflammation, cognitive impairment, or chronic stress. These models are valuable because they link microbial changes with disease-relevant outcomes, but they are also heterogeneous and often differ in intervention type, dose, and microbiota methodology. Their main contribution is therefore to show that metabolic, immune, or inflammatory disruption can modify the EVOO–microbiota relationship.

In a rat model of high-fat/high-fructose diet-induced metabolic syndrome, EVOO supplementation partially reversed dysbiosis, increasing Bifidobacteriaceae/*Bifidobacterium*, Eubacteriaceae, Anaeroplasmataceae, and Ruminococcaceae-related taxa, while reducing Enterococcaceae/*Enterococcus*, Staphylococcaceae/*Staphylococcus*, Corynebacteriaceae, and *Bilophila*. These changes occurred alongside lower body-weight gain and improved insulin sensitivity and lipid-related outcomes [68]. Similarly, in non-obese diabetic (NOD) mice, EVOO increased the Bacteroidetes/Firmicutes ratio and taxa related to SCFA production, including *Lachnoclostridium* and *Ruminococcaceae_UCG-005*, in parallel with delayed type 1 diabetes progression and improved glucose-related outcomes [69]. These findings fit with the broader idea that EVOO may interact with microbial pathways involved in glucose metabolism, low-grade inflammation, and immune regulation. However, the taxa involved differ between models, indicating that the response depends on the underlying metabolic and immune disturbance.

Cardiovascular and neuroimmune models extend this concept beyond classical metabolic disease. In spontaneously hypertensive rats, an EVOO-enriched diet was associated with fecal microbiota changes detected by DGGE/qPCR and lower systolic blood pressure; clostridia cluster XIV was inversely correlated with systolic blood pressure [70]. Although this approach has lower taxonomic resolution than current sequencing methods, it links EVOO-associated microbial changes with a cardiovascular phenotype. Similarly, in experimental autoimmune encephalomyelitis (EAE) in Dark Agouti rats, EVOO, oleic acid, and HT reduced lipopolysaccharide (LPS) and lipopolysaccharide-binding protein (LBP) in the brain, spinal cord, and blood, together with oxidative stress and inflammatory markers [71]. This places EVOO and its major bioactive components within a gut-derived microbial-product axis relevant to endotoxemia and neuroinflammation. However, because microbiota composition was not assessed, this study should be interpreted as evidence for gut-derived inflammatory signaling, not for direct microbiota remodeling.

Gut–brain models provide a similar message, but with different levels of mechanistic support. In chronic unpredictable mild stress-induced rats, olive oil modified fecal microbiota and neurotransmitter-related metabolism and reduced selected inflammatory markers, although fish oil produced clearer behavioral and barrier-protective effects [72]. This suggests that chronic stress is a context in which dietary lipid quality can shape gut–brain-related readouts, while also showing that olive oil is not necessarily the most effective lipid comparator for depression-like outcomes. A more direct microbiota–brain link was reported in AlCl_3_-induced mild cognitive impairment, where EVOO improved cognitive performance, reduced brain inflammatory and oxidative markers, and modified fecal microbiota compared with AlCl_3_-treated rats [73]. The EVOO group showed higher Bacteroidetes, Bacteroidia, and Bacteroidales and lower Clostridia/Clostridiales; at the genus level, EVOO increased *Alistipes*, *Odoribacter*, and *Parabacteroides*, while reducing the Prevotellaceae_NK3B31_group and the Prevotellaceae_UCG-001 group. These taxa should be interpreted within the disease model rather than as universal markers of EVOO action, since their relevance derives from their association with cognitive, inflammatory, and oxidative readouts in this experimental setting.

Human data also suggest that baseline health status influences whether diet-related microbial changes are detectable. In CORDIOPREV, long-term Mediterranean and low-fat dietary interventions partially restored gut microbiota dysbiosis in obese patients with severe metabolic dysfunction, whereas obese or non-obese subjects without marked metabolic impairment showed no relevant microbiota changes [74]. This does not isolate olive oil as an active component, but it supports the concept that a disturbed metabolic background may create a more responsive microbial ecosystem. The same caution applies to neurological and renal clinical contexts. In pediatric epilepsy, an olive oil-rich Mediterranean ketogenic diet produced microbiota and SCFA-related changes, but these effects cannot be separated from ketosis, carbohydrate restriction, or other dietary components [75]. In CKD patients undergoing hemodialysis, 40 mL/day EVOO for 14 days increased fecal SCFA, although taxonomic microbiota profiling was not performed [56]. These studies reinforce host context as an important modifier of the response, while also showing that microbiota composition, microbial metabolites, and clinical outcomes should not be conflated.

Across the available evidence, disease-specific models indicate that the EVOO–microbiota relationship depends strongly on host physiology. Metabolic dysfunction, immune activation, neuroinflammation, stress, and renal disease may all condition the magnitude and direction of the response. However, the endpoints used across studies differ substantially: some assess microbiota composition, others measure microbial products such as LPS/LBP, and others focus mainly on SCFA or inflammatory markers. For this reason, the evidence supports host context as a determinant of the EVOO response, but it does not justify treating compositional microbiota data, microbial-product markers, and host inflammatory outcomes as interchangeable endpoints.

### 5.4. Olive-Derived Phenolics and Microbial Biotransformation

Among the different minor bioactive constituents present in VOO and EVOO, the phenolic fraction offers the strongest mechanistic evidence explaining why EVOO may differ from ROO. Olive phenolics may reach the colon, where they can be transformed by microbial enzymes into smaller phenolic metabolites. However, the bioavailability and chemical form of olive-derived phenolics reaching the colon are strongly influenced by intestinal and hepatic phase I/II metabolism. In the small intestinal mucosa, secoiridoids and simple phenolics undergo extensive biotransformation during enterocyte absorption, including reduction and phase II conjugation (glucuronidation and sulfation), resulting in a range of metabolites with altered physicochemical properties and bioactivity [19,76]. Studies using intestinal perfusion and Caco-2 models have demonstrated that olive oil secoiridoids are extensively reduced and glucuronidated during transfer across the jejunum and ileum, leading to conjugated metabolites as the predominant forms entering portal circulation. HT and tyrosol are also subject to extensive phase II metabolism, with sulfation and glucuronidation representing the main circulating forms after dietary intake [77]. In addition, secoiridoid precursors such as oleuropein derivatives undergo partial hydrolysis and structural transformation in the gastrointestinal tract prior to absorption [78]. These conjugated metabolites may be secreted into bile and re-enter the intestinal lumen through enterohepatic circulation, thereby contributing to repeated intestinal exposure [79]. Although direct evidence for olive oil phenolics remains limited, studies on other dietary polyphenols, including resveratrol and flavonoids, have shown that bacterial deconjugating enzymes, bacterial deconjugating enzymes, particularly β-glucuronidases and potentially sulfatases, can hydrolyze glucuronide and sulfate conjugates, regenerating free phenolic compounds that may undergo reabsorption or further microbial biotransformation and interact with the gut microbiota and intestinal epithelium [79,80,81]. These findings support the concept that enterohepatic recirculation, together with microbial deconjugation, may prolong intestinal exposure to phenolic metabolites and thereby contribute to their local biological activity [19,82].

Once phenolic metabolites reach the colonic environment, they become substrates for microbial enzymatic transformation and fermentation processes. In a study using phenolic leaf extracts rich in oleuropein with EVOO included as a reference matrix, Rocchetti et al. showed that during simulated gastrointestinal digestion and fecal fermentation, oleuropein decreased while HT and other low-molecular-weight phenolic metabolites increased; fermented EVOO also showed increased HT, indicating that EVOO secoiridoids can contribute to colonic phenolic metabolite formation. The microbial effects were particularly evident for Coriobacteriaceae and *Collinsella*, taxa relevant to polyphenol and bile acid metabolism [83]. The same substrate–microbe logic has been observed with other olive-derived phenolics. Purified oleocanthal, a characteristic EVOO secoiridoid, was rapidly transformed in fecal fermentation systems into oleoglycine, tyrosol acetate, and tyrosol [84], while simple olive phenolic alcohols also showed lower-gut availability and microbial transformation [85]. Using a simulated gastrointestinal–colon model with pooled human fecal suspension, HT and tyrosol generated compartment- and time-dependent metabolite profiles, with a broader range of putative metabolites for HT than for tyrosol, and both parent compounds remaining detectable after 24 h of colonic incubation [85]. Consistently, simulated digestion and fecal fermentation of HT showed that this compound can reach the colon and be transformed into phenolic- and indole-related metabolites, while increasing total SCFA production [86]. This study supports HT as a microbiota-accessible olive phenolic, although the model was in vitro and did not test whole EVOO. Related phenolipid studies further support the concept that olive-derived simple phenols can be delivered to the lower gut in esterified forms and subsequently released by microbial enzymes. Tyrosol long-chain fatty acid esters reached the cecum and colon in mice and were hydrolyzed by fecal microbiota and by *Lactobacillus johnsonii*, *L. reuteri*, and *L. gasseri*, whereas free tyrosol was rapidly absorbed in the small intestine [87]. In a complementary in vitro approach, synthetic HT-SCFA and tyrosol-SCFA acyl esters were also hydrolyzed by digestive enzymes, fecal microbiota, and *Lactobacillus* strains, releasing both olive phenolics and SCFAs in a structure-dependent manner [88]. These phenolipid models support microbial enzymatic release of olive-phenolic derivatives, but their relevance to naturally consumed EVOO remains indirect.

Olive by-products provide a related line of evidence since they deliver phenolics within fiber-rich or semi-solid matrices. Olive pâté, an EVOO-production by-product, increased Lactobacillaceae and Bifidobacteriaceae in a Simulator of the human intestinal microbial ecosystem (SHIME) model and underwent microbial transformation of HT-related compounds [89]. Similarly, a biologically debittered olive patè enriched with *Lactiplantibacillus plantarum* modulated human colonic microbiota in an ex vivo fermentation model and showed potential prebiotic, eubiotic, and bifidogenic activity [90]. Compositional studies of olive pomace powders support the plausibility of this mechanism, showing that olive by-products can provide HT, tyrosol derivatives, and phenolics bound to dietary fiber [91]. Compared with free phenolics, fiber-bound phenolics are generally less accessible for small-intestinal absorption and may therefore reach the colon, where microbial enzymes can release and transform them into smaller phenolic metabolites. In humans with mild hypercholesterolemia, olive pomace-enriched biscuits did not substantially alter alpha or beta diversity, but showed trends toward increased *Bifidobacterium* and increased phenolic metabolites such as 3,4-dihydroxyphenylacetic acid (DOPAC) and homovanillic acid [92]. Taken together, these findings suggest that olive-derived phenolic-rich substrates may participate in microbial and host phenolic metabolism even when broad community restructuring is limited.

Isolated phenolic studies extend this mechanism into metabolic and inflammatory settings, but their interpretation depends strongly on the disease model and whether the compound is administered alone or combined with other interventions. In an HFD model, tyrosol partially normalized dysbiosis, including modulation of the F/B balance and increased Verrucomicrobia, alongside improvements in obesity-related metabolic outcomes and thermogenic markers [93]. HT was also reported to counteract PM2.5-induced metabolic dysfunction while restoring microbial richness [94]. This microbiota-associated antioxidant effect is also supported in an acute oxidative stress model, where oral HT modified colonic microbiota structure, partially counteracted diquat-induced changes in Firmicutes, Bacteroidetes, and Parabacteroides, restored colonic butyrate, and activated Nuclear factor erythroid 2-related factor 2 (Nrf2)-related antioxidant responses. However, this remains evidence for isolated HT rather than whole EVOO, and the study does not demonstrate a direct barrier-protective effect because jejunal and ileal morphology were not significantly modified [95]. In aged mice exposed to a traumatic-stress paradigm, HT reduced anxiety-like responses and neuroinflammation while preserving gut microbiota composition, supporting a possible microbiota–gut–brain contribution of this olive-derived phenolic in stress resilience [96]. A further strain-level perspective comes from work showing that dietary exposure to different fats, including EVOO, may influence phenotypic traits of *Enterococcus* isolates, suggesting that olive oil-related effects may occur not only at the level of community composition but also at the level of within-genus functionality [97].

The dextran sodium sulfate (DSS) colitis models deserve separate consideration because they connect olive-derived phenolics with microbiota, epithelial barrier integrity, and intestinal inflammation. Across these models, HT consistently linked microbiota remodeling with recovery of SCFA-related outputs. One study reported restored microbial diversity, a shift from potentially pathogenic taxa toward SCFA-producing genera, and increased fecal acetate, propionate, and butyrate [98], whereas another found partial restoration of microbial diversity and SCFA production, reduced inflammation-associated taxa such as Bacteroidaceae, Desulfovibrionaceae, and *Ruminococcus*, and increased SCFA-associated taxa such as Lachnospiraceae, Muribaculaceae, ASF356, and *Colidextribacter* [99]. Oleuropein also showed microbiota-related protection in DSS colitis, increasing alpha diversity and enriching *Lactobacillus*, *Turicibacter*, *Alistipes*, *Bifidobacterium*, and *Ruminococcus*, while modulating bile acid metabolism [100]. A related but more complex model was reported by Yu and coworkers [101], where tyrosol combined with *Lactobacillus plantarum* SC-5 increased *Lactobacillus, Bifidobacterium*, and *Akkermansia*, reduced Proteobacteria/*Proteus*, and transferred part of the protective effect through fecal microbiota transplantation (FMT). Because this intervention combined tyrosol with a probiotic, the effect cannot be attributed to tyrosol alone; however, it supports the broader principle that olive-derived phenolics may interact with microbial remodeling to attenuate experimental colitis.

Although VOO and EVOO also contain other minor bioactive constituents, including tocopherols, phytosterols, triterpenes, and squalene, the available mechanistic evidence linking these compounds to gut microbiota modulation remains limited. In contrast, olive-derived phenolics have been much more extensively investigated because they undergo microbial biotransformation in the gastrointestinal tract, generating metabolites capable of interacting with both the gut microbiota and the host. Therefore, this section focuses primarily on phenolic compounds, for which the current evidence is substantially more robust.

In conclusion, mechanistic studies support a bidirectional model: olive-derived phenolics are transformed by gut microbes into smaller metabolites, and these compounds may in turn modulate microbial ecology and function. This provides a plausible explanation for differences between EVOO and ROO. This evidence explains how the minor fraction of EVOO may interact with the gut ecosystem, while also highlighting that whole-oil interventions are still needed to determine the physiological relevance of these mechanisms in vivo. Studies addressing olive-derived phenolics, by-products, and microbial biotransformation are summarized in Table 4.

### 5.5. Mediterranean Diet Context: Olive Oil as Part of a Dietary Pattern

A substantial part of the human evidence comes from MD studies in which olive oil is a central lipid source. These studies are highly relevant because they reflect real dietary exposure, but they cannot isolate the contribution of olive oil from other microbiota-active components of the diet, including fiber, legumes, fruits, vegetables, nuts, fish, and other polyphenol sources. In this context, the MD provides a combined ecological pressure on the gut microbiota: olive oil contributes oleic acid and phenolic compounds, while plant foods provide fermentable substrates and additional polyphenols. Therefore, these studies are best interpreted as evidence for a dietary pattern in which olive oil is a key component.

The CORDIOPREV studies illustrate this point particularly well. In obese men with coronary heart disease, both an MD rich in olive oil and a low-fat/high-complex-carbohydrate diet modulated gut microbiota and improved insulin sensitivity, while the MD specifically reduced *Prevotella* and increased *Roseburia* and *Oscillospira* [102]. In a later CORDIOPREV analysis, the response appeared to depend strongly on baseline metabolic status. Obese subjects showed a dysbiotic profile characterized by lower Actinobacteria and Bacteroidetes, a higher F/B ratio, and reduced *Bacteroides*, *Prevotella*, *Roseburia*, *Faecalibacterium*, and *Ruminococcus*. After two years, both Mediterranean and low-fat diets increased *Bacteroides, Prevotella*, and *Faecalibacterium*, whereas the MD additionally increased Roseburia, Ruminococcus, *Parabacteroides distasonis*, and *Faecalibacterium prausnitzii* [74]. This suggests that the MD may contribute to the restoration of saccharolytic and potentially butyrate-associated taxa, especially when a dysbiotic metabolic phenotype is present at baseline.

Evidence from observational and shorter-term dietary studies is consistent with this pattern-based interpretation. In adults without declared pathology, higher adherence to a Mediterranean dietary pattern was associated with higher Bacteroidetes, Prevotellaceae, and *Prevotella*, lower Firmicutes and *Lachnospiraceae*, and higher fecal propionate and butyrate concentrations among subjects with an MD Score ≥ 4 [103]. Similarly, a higher MD adherence and greater plant-food intake were associated with higher fecal SCFA and taxa such as *Prevotella*, *Lachnospira*, and *Roseburia* [13]. These findings reinforce the idea that olive oil acts within a diet rich in fermentable substrates and plant polyphenols, rather than as the sole driver of the microbial response. Mediterranean dietary exposure has also been linked with oleic acid-derived endocannabinoidome mediators, including oleoylethanolamide and 2-oleoylglycerol, and microbiota-related features, suggesting that the lipid component of the diet may interact with host lipid signaling and gut microbial ecology [104]. A more EVOO-focused, but still pattern-based, intervention using an MD supplemented with 40 g/day high-quality EVOO for 3 months reported increased lactic acid bacteria and improved oxidative and inflammatory markers in subjects with overweight or obesity [105]. In another clinical context, an olive oil-rich Mediterranean ketogenic diet in pediatric epilepsy achieved high adherence and clinical response, while inducing genus-level microbial shifts without significant alpha- or beta-diversity changes and with broadly stable major fecal SCFA concentrations; however, because olive oil contributed almost 60% of total fat within a ketogenic diet, the effects cannot be separated from ketosis, carbohydrate restriction, or other dietary components [75].

Overall, MD studies provide the most translationally relevant context for olive oil exposure, but also the least oil-specific one. They suggest that olive oil may contribute to a dietary environment favoring saccharolytic, SCFA-associated, and anti-inflammatory microbial profiles, particularly in individuals with metabolic dysfunction. However, the observed microbiota effects should not be attributed exclusively to olive oil unless the study design includes appropriate oil-specific comparators. In this setting, olive oil is best interpreted as a central component of a broader dietary network, acting together with fiber, plant polyphenols, and other MD features to shape microbial ecology and microbial metabolism. Human studies evaluating olive oil, olive oil-rich dietary patterns, and related microbiota or systemic outcomes are summarized in Table 5.

## 6. Olive Oil and Intestinal Barrier Function

The intestinal barrier is a key physiological interface through which olive oil may influence host responses beyond changes in microbial composition. Barrier function should be evaluated through specific endpoints, including epithelial permeability, tight-junction integrity, mucosal injury, and systemic exposure to gut-derived microbial products such as LPS or LBP. The available evidence is still mostly preclinical, but it suggests that EVOO and olive-derived phenolics may act on barrier integrity under inflammatory or metabolic stress.

The most direct whole-oil evidence comes from DSS colitis, where daily oral administration of EVOO from Apulian cultivars attenuated intestinal injury, reducing body-weight loss and rectal bleeding, improving histological damage, and lowering fluorescein isothiocyanate (FITC)-dextran permeability [110]. Because FITC-dextran reflects paracellular permeability, this study provides direct evidence that whole EVOO can protect barrier function under acute inflammatory stress. The cultivar-dependent response also suggests that EVOO composition may influence the magnitude of this effect. In the same DSS-colitis framework, studies with olive-derived phenolics provide more detailed epithelial and microbiota-related endpoints. Oleuropein restored tight-junction markers such as zonula occludens (ZO)-1 and claudin-3, while FMT and hyodeoxycholic acid (HDCA) experiments supported a microbiota–bile acid–barrier mechanism [100]. HT also improved barrier-related endpoints, increasing goblet-cell preservation, Muc2 expression, and tight-junction proteins, including claudin-1, occludin and ZO-1 [99]. A related DSS model using tyrosol combined with *Lactobacillus plantarum* SC-5 preserved overlapping tight-junction markers, including ZO-1, occluding, and claudin-3, and transferred part of the protective phenotype through FMT [101]. This epithelial-protective signal is also supported outside DSS colitis. In diquat-challenged piglets and IPEC-J2 cells, HT preserved intestinal morphology and tight-junction proteins, reduced permeability-related damage, and improved antioxidant defenses through PI3K/Akt-Nrf2 signaling and mitophagy [111]. Because microbiota and SCFA changes were limited, this study supports epithelial redox protection more strongly than microbiota-mediated barrier modulation. In an EAE mouse model, oleacein reduced colon and ileum FITC-dextran permeability; lowered serum intestinal fatty acid-binding protein (iFABP) and sCD14; preserved colonic mucin staining, galectin-3, and glial cell line-derived neurotrophic factor (GDNF); and attenuated tumor necrosis factor alpha (TNFα)-induced barrier dysfunction in Caco-2 monolayers [112]. Additional in vitro evidence adds a possible metabolic link: after fecal fermentation, HT-derived and tryptophan-related metabolites showed predicted binding affinity for AhR, a receptor involved in epithelial homeostasis and barrier repair [86]. However, not all preclinical studies consistently support a direct barrier-protective effect of olive oil. Thomas et al. [61] showed that olive oil reduced serum endotoxin in HFD-fed mice. However, unlike perilla oil, olive oil did not significantly upregulate colonic claudin-1, ZO-1, or MUC-1 expression. Therefore, this study supports reduced exposure to gut-derived inflammatory products after olive oil supplementation, but it does not provide strong evidence for direct restoration of epithelial tight-junction integrity in this model [61]. Barrier-related findings from the same *Muc2*^−/−^ model add an important nuance. The corn oil diet increased FITC-dextran permeability, whereas the Mediterranean-like and milk-fat diets showed higher intestinal alkaline phosphatase activity than the olive oil and corn oil diets. Therefore, this study does not support a simple barrier-protective effect of olive oil alone; rather, it indicates that epithelial and mucosal-defense outcomes may depend on the broader fatty acid blend and on host barrier vulnerability [66]. Overall, these phenolic studies suggest that olive-derived compounds may influence barrier integrity through converging mechanisms involving tight-junction preservation, mucus-related pathways, epithelial redox control, microbiota-derived metabolites, bile acid signaling, and AhR-related epithelial homeostasis.

Human evidence is not direct and comes mainly from postprandial studies measuring circulating LPS. In patients with metabolic syndrome, phenol-rich VOO attenuated the postprandial rise in plasma LPS [107]. Similarly, in patients with impaired fasting glucose, adding 10 g of EVOO to a meal blunted the postprandial increase in serum LPS [108]. This postprandial endotoxemia effect was extended in impaired fasting glucose patients by showing that EVOO added either to a Mediterranean-type meal or to chocolate reduced not only circulating LPS but also serum zonulin, an indirect marker of gut permeability. The same study linked lower LPS and zonulin with improved postprandial glucose, insulin, and glucagon-like peptide-1 (GLP-1) responses, supporting the idea that EVOO may attenuate metabolic endotoxemia together with changes in gut permeability-related signaling [109]. Although these human studies do not measure epithelial permeability directly, the combined reduction in circulating LPS and zonulin supports their interpretation as evidence for reduced postprandial endotoxemia with accompanying improvement in gut permeability-related markers.

The evidence reviewed here suggests that EVOO and olive-derived phenolics may help preserve epithelial integrity and reduce exposure to microbial inflammatory products, although studies using non-EVOO olive oil diets indicate that barrier-related effects may depend on the broader fatty acid matrix and host context. Whole EVOO evidence is strongest for DSS-related permeability and mucosal injury. However, current clinical evidence should be interpreted as supporting a potential modulation of barrier-related processes rather than providing direct evidence of improved intestinal permeability. To date, direct assessment of intestinal permeability using established tests such as the lactulose–mannitol assay has not been performed in olive oil intervention studies. Therefore, current evidence should be interpreted as indicative of barrier modulation rather than direct measurement of intestinal permeability. Future studies should combine well-characterized EVOO interventions with direct permeability assays, tight-junction markers, mucus-layer assessment, microbial metabolites, and LPS/LBP to clarify whether the observed effects are mediated by the whole-oil matrix, olive-derived phenolics, microbiota-derived metabolites, or reduced inflammatory injury.

## 7. Olive Oil and Inflammatory Bowel Diseases

Inflammatory bowel diseases (IBD) are characterized by chronic relapsing intestinal inflammation in which diet, gut microbiota, epithelial barrier integrity, and mucosal immune responses interact within the intestinal environment. Dietary modulation of microbiota is therefore relevant to IBD, although current evidence does not yet support specific dietary patterns or food constituents that consistently define microbiota profiles or disease markers in patients with IBD. Within this context, EVOO and olive-derived phenolics have mainly been evaluated in experimental colitis models, where they may influence colitis-related processes such as epithelial damage, microbial metabolism, oxidative stress, and cytokine signaling [113].

Whole-oil evidence is available from the study by Cariello et al. [110]. Daily oral administration of EVOO attenuated several features of acute colitis, including body-weight loss, rectal bleeding, intestinal permeability, and histological damage. Colonic inflammatory gene expression was also reduced, with cultivar-dependent differences in the magnitude of the response. These findings support an anti-colitic effect of whole EVOO in an acute inflammatory model, while also indicating that EVOO composition may influence biological activity. Evidence from spontaneous colitis models adds a different perspective to the DSS-based whole EVOO data. In *Muc2*^−/−^ mice, a model of mucus barrier deficiency and microbiota-dependent spontaneous colitis, the most consistent protection was observed with a Mediterranean-like fat blend rather than with olive oil alone. This blend reduced disease activity, histological damage, ulceration, and crypt abscesses, and promoted immune-repair pathways involving tolerogenic CD103+CD11b+ dendritic cells and Th22/IL-17+IL-22+ cell populations. The olive oil diet showed partial benefits, including lower disease activity than milk fat, but it did not reproduce the full protective profile of the Mediterranean-like fat blend. Therefore, this study supports the relevance of lipid quality in colitis, while indicating that the protective effect may depend on a balanced fat pattern rather than on olive oil as a single fat source [66]. Studies with olive-derived phenolics provide more mechanistic detail. Oleuropein reduced DSS-induced disease activity, colon shortening, inflammatory infiltration, cytokine production, myeloperoxidase (MPO), and malondialdehyde (MDA), while improving tight-junction proteins and suppressing nuclear factor kappa B (NF-κB) signaling [100]. The same study linked protection to bile acid metabolism, particularly HDCA, and supported this mechanism through FMT and HDCA validation experiments [100]. Two DSS-colitis studies support HT as a relevant olive-derived phenolic in experimental intestinal inflammation. Miao [98] showed that HT reduced body-weight loss, disease activity index (DAI), colon shortening, histological damage, and epithelial apoptosis, while improving antioxidant defenses, inhibiting NLRP3 inflammasome activation, and restoring gut microbiota diversity and SCFA levels. Wang et al. [99] reported a complementary mechanism, with HT reducing colitis severity, MPO activity, and pro-inflammatory cytokines, increasing IL-10, activating Nrf2/heme oxygenase-1 (HO-1) signaling, inhibiting Toll-like receptor 4 (TLR4)/p65 NF-κB activation, and improving mucus and tight-junction markers. Together, these studies indicate that HT can attenuate experimental colitis through convergent antioxidant, anti-inflammatory, barrier-related, and microbiota-associated mechanisms. This mechanistic line is extended by the tyrosol–*Lactobacillus plantarum* SC-5 study. In DSS colitis, the combined treatment reduced disease activity, histological damage, oxidative stress, and inflammatory cytokines, while preserving epithelial junction proteins and inhibiting NF-κB/mitogen-activated protein kinase (MAPK) signaling [101]. The FMT experiment, in which microbiota from treated mice transferred part of the protective effect, strengthens the idea that phenolic-associated protection can involve microbiota-dependent mechanisms.

Taken together, IBD-related studies suggest protective effects of whole EVOO and olive-derived phenolics in experimental colitis. The most consistent outcomes are reduced epithelial damage, lower inflammatory signaling, improved tight-junction integrity, and reduced oxidative stress. However, translation to human IBD remains unresolved. Future studies should test well-characterized EVOO interventions in patients with IBD and include clinical activity, fecal calprotectin, endoscopic or histological endpoints, permeability markers, cytokines, bile acids, SCFA, and phenolic metabolites. Thus, the current evidence supports biological plausibility in intestinal inflammation, but not clinical efficacy in patients with IBD.

## 8. Interactions with Other Health Systems

### 8.1. Gut–Brain Axis: Potential Effects on Cognitive Health and Neuroinflammation

Evidence for a gut–brain role of olive oil remains suggestive, and most available studies do not demonstrate a direct microbiota-mediated mechanism [18]. At the preclinical level, initial mechanistic support comes from models where olive oil or olive-derived compounds influence neuroinflammatory pathways linked to gut-derived microbial products. As already discussed in relation to barrier function, authors showed that EVOO, oleic acid, and HT reduced LPS and LBP in blood, brain, and spinal cord in EAE, together with lower oxidative and inflammatory markers. Although microbiota composition was not assessed, the reduction in LPS/LBP places EVOO and its major bioactive components within a gut-derived inflammatory axis that can influence neuroinflammatory injury [71]. This gut-derived inflammatory axis is further supported by isolated-compound evidence with oleacein in EAE [112]. In this model, oleacein improved EAE-associated intestinal barrier dysfunction and reduced colonic inflammatory and oxidative alterations, while producing only limited microbiota changes, mainly increased Akkermansiaceae abundance [112]. In AlCl_3_-induced mild cognitive impairment, EVOO also improved learning and memory performance and reduced brain pro-inflammatory cytokines and oxidative stress markers [73]. This study adds a more cognition-focused model to the neuroinflammatory evidence, although the causal contribution of microbiota changes remains unresolved. Stress-related models provide a more cautious picture. In chronic stress paradigms, olive oil modified gut–brain-related biological responses, including neurotransmitter-related metabolism, microbiota-associated readouts, and inflammatory markers, but fish oil produced clearer behavioral protection [64,72]. A similar caution applies to obesogenic high-fat models focused on cognitive or neuroinflammatory outcomes. In C57BL/6 mice fed 45–fat diets differing in fatty acid source, the olive oil/MUFA-rich diet did not show a clear gut–brain protective profile compared with the *n*-3 polyunsaturated fatty acid (PUFA) diet; instead, it was associated with increased inflammatory signaling in brain and intestine under high-fat conditions. Together with recent evidence from an EVOO-enriched HFD model of scopolamine-induced Alzheimer’s-like alterations, these findings indicate that lipid quality and oil matrix can modulate neuroinflammatory outcomes and that dose and dietary background strongly condition gut–brain responses [114,115].

Human evidence is more limited and mainly observational. Higher habitual intake of VOO or EVOO was associated with more favorable cognitive trajectories, whereas common olive oil showed less favorable associations [33]. In a different neurological context, an olive oil-rich Mediterranean ketogenic diet in pediatric epilepsy achieved high adherence and clinical response together with microbiota and SCFA-related changes, supporting the relevance of olive oil-rich dietary patterns in gut–brain settings, but not isolating olive oil as the causal component [75]. Human fatty acid substitution studies further support the relevance of the oleic acid–palmitic acid balance for neuroimmune outcomes. In young adults, replacing a high-palmitic acid dietary pattern with a low-palmitic/high-oleic acid diet modified brain activation during cognitive testing and reduced inflammatory responses of LPS-stimulated immune cells [116]. A similar crossover study in older adults showed that decreasing dietary palmitic acid while increasing oleic acid altered prefrontal activation and salience-network connectivity, together with lower LPS-stimulated production of IL-1β, IL-6, and IL-8 by peripheral immune cells [117]. These data support a biologically coherent link between oleic acid-rich lipid exposure, peripheral inflammatory tone, and brain function.

Altogether, the gut–brain evidence suggests a plausible link between olive oil quality, gut-derived inflammatory products, and neurocognitive or neuroinflammatory outcomes. However, the current evidence remains indirect: preclinical studies support mechanisms involving LPS/LBP and neuroinflammation, whereas human data are observational or based on fatty acid substitution rather than EVOO itself. Therefore, a microbiota-mediated cognitive effect of EVOO cannot yet be established.

### 8.2. Metabolic Impact: Metabolic Syndrome, Insulin Resistance, and Postprandial Metabolism

The metabolic evidence is stronger than the gut–brain evidence, but it remains highly dependent on study design. EVOO has been tested in chronic interventions, acute postprandial studies, and animal models of metabolic dysfunction. The relevant outcomes include LDL-c, HbA1c, insulin sensitivity, postprandial lipemia, body adiposity, glucose-related outcomes, and postprandial lipoprotein metabolism.

Preclinical evidence indicates that EVOO, olive oil, and olive-derived compounds can influence metabolic outcomes under conditions of diet-induced metabolic stress, although the direction and magnitude of the response depend on the oil matrix, model, and comparator fat. In HFD models, olive oil-based diets have been associated with metabolic profiles distinct from saturated fat-rich diets, including lower adiposity compared with palm oil [58]. Consistently, in mice fed HFD based on different oils, the olive oil group showed lower serum triglycerides and lower serum and hepatic MDA than the lard-oil group, although the high-fat olive oil diet still induced dyslipidemic and hepatic lipogenic changes compared with a normal diet [59]. At recommended intake, olive oil also improved insulin sensitivity/signaling, fatty acid oxidation, and hepatic metabolic outcomes compared with coconut oil, supporting the metabolic relevance of replacing saturated fat-rich oils with MUFA-rich olive oil in preclinical models [60]. In the comparative HFD model by Thomas et al. [61], partial replacement of lard with olive oil also reduced body-weight gain, liver weight, epididymal fat weight, and serum leptin, and downregulated hepatic and adipose lipogenic genes, although perilla oil showed a clearer induction of hepatic β-oxidation-related genes, and perilla and safflower oils showed stronger triglyceride-lowering effects. However, this MUFA-centered interpretation is not universal across disease contexts. In a colitis-prone *Muc2*^−/−^ model, glucose clearance was more favorable with a Mediterranean-like fat blend and with milk fat than with olive oil or corn oil, despite all diets being isocaloric and matched for total fat content. This suggests that, when mucus barrier dysfunction and intestinal inflammation are present, metabolic regulation may depend on the complete fatty acid matrix rather than on olive oil-derived MUFA enrichment alone [66]. In diet-induced metabolic syndrome, EVOO modulated microbial and metabolic parameters, supporting a link between oil quality, host metabolic state, and gut-associated metabolic regulation [68]. In NOD mice, EVOO was associated with delayed type 1 diabetes progression and improved glucose-related outcomes, alongside changes in taxa related to SCFA production [69]. Evidence from isolated compounds is also supportive, where tyrosol improved obesity-related metabolic outcomes and thermogenic markers in HFD-fed mice [93].

In humans, the most direct evidence comes from interventions comparing oils with different degrees of refinement or phenolic content. In patients undergoing coronary angiography, 25 mL/day of polyphenol-rich EVOO for 6 weeks reduced total cholesterol and LDL-C compared with the same dose of ROO, while other lipid fractions were not significantly modified [106]. This trial is relevant for metabolic interpretation because EVOO and ROO were compared under matched daily oil intake, allowing lipid effects to be considered in relation to oil quality rather than total added fat. Human postprandial studies point to a complementary mechanism. In impaired fasting glucose, adding 10 g EVOO to a meal blunted the postprandial increase in apolipoprotein (apo)-B48 and oxidized LDL [108]. In the same postprandial metabolic context, EVOO also reduced the rise in glucose while enhancing insulin and GLP-1 responses in impaired fasting glucose patients, with parallel reductions in circulating LPS and zonulin. These findings integrate EVOO’s effects on glycemic control with reduced gut permeability-related endotoxemia, rather than treating postprandial metabolism and barrier function as separate mechanisms [109]. These data position EVOO within postprandial metabolic regulation, where chylomicron handling, postprandial lipoprotein metabolism, and oxidative modification of circulating lipids are relevant outcomes.

MD studies provide translational support. An MD rich in olive oil improved insulin sensitivity in men with coronary heart disease [102], and longer-term analyses showed that metabolic status influenced the microbiota response to dietary intervention [74]. Luisi et al. [105] similarly reported beneficial systemic effects after an MD supplemented with high-quality EVOO. Controlled fatty acid substitution studies further support the relevance of the dietary lipid profile for metabolic inflammation. Lowering the dietary palmitate-to-oleate ratio did not significantly improve hepatic or peripheral insulin sensitivity in healthy young adults [118]. This negative result suggests that increasing oleic acid at the expense of palmitic acid may not be sufficient to reproduce the metabolic effects attributed to EVOO or Mediterranean dietary patterns, particularly in metabolically healthy individuals.

In summary, EVOO may influence metabolic health through effects on lipid profile, glucose-related outcomes, adiposity in experimental models, and postprandial lipoprotein metabolism. The most direct human evidence supports modest lipid improvements with polyphenol-rich EVOO compared with ROO, while MD studies support metabolic benefits in cardiometabolic populations without isolating the specific contribution of EVOO.

### 8.3. Immune System Modulation: Regulation of Systemic Inflammatory Responses

Olive oil-related immune effects are supported by both whole-oil studies and phenolic-focused models. The most relevant endpoints are C-reactive protein (CRP), cytokine production, LPS-stimulated immune responses, NF-κB/MAPK signaling, and oxidative inflammatory markers.

Preclinical models suggest that olive oil-related interventions can regulate inflammatory pathways at the intestinal and systemic levels. As discussed in the IBD section, whole EVOO reduced colonic expression of inflammatory genes in DSS colitis [110], while oleuropein reduced TNF-α, IL-1β and IL-6 and inhibited NF-κB signaling [100]. HT also reduced DSS-induced inflammatory activation through mechanisms involving NLRP3 inflammasome inhibition and Nrf2/NF-κB modulation [98,99]. A tyrosol–*Lactobacillus plantarum* SC-5 intervention also reduced cytokine production, oxidative stress, and NF-κB/MAPK activation in DSS colitis [101]. These findings support local immune regulation in experimental intestinal inflammation. In HFD-fed mice, EVOO also reduced hepatic LBP and increased mucosal immune-regulatory markers compared with lard, linking the dietary lipid source with microbial-product exposure and mucosal immune tone [62]. Thomas et al. [61] further supports an anti-inflammatory effect of olive oil under HFD-induced intestinal stress: olive oil reduced colonic IL-6 protein levels, lowered colonic TNF-α, IL-6, and IL-1β mRNA expression, increased IL-10 mRNA expression, and reduced p-p65, inducible nitric oxide synthase (iNOS), and cyclooxygenase-2 (COX-2) protein expression compared with HFD. Nevertheless, the overall anti-inflammatory response was more pronounced with perilla oil, indicating that the magnitude of immune modulation may differ among unsaturated vegetable oils.

Human studies provide more direct evidence for systemic inflammatory modulation. In patients undergoing coronary angiography, polyphenol-rich EVOO reduced CRP compared with ROO after 6 weeks and increased ex vivo LPS-stimulated IL-10 production, whereas LPS-stimulated IL-6 was not significantly modified [106]. This suggests that EVOO may influence inflammatory tone and anti-inflammatory cytokine responsiveness under cardiovascular risk conditions. In a separate pre–post intervention, polyphenol-rich EVOO was associated with reduced salivary IL-1β, together with metabolic changes [55], although the absence of a control group limits causal interpretation. Acute postprandial studies further indicate that EVOO-related immune effects may emerge under metabolic challenge. In metabolic syndrome, phenol-rich VOO reduced postprandial TLR4/NF-κB-related inflammatory activation, including lower TLR4, SOCS3, NF-κB activation and pro-inflammatory gene expression [107]. In impaired fasting glucose, EVOO blunted the postprandial rise in LPS-associated oxidative stress, reducing NADPH oxidase 2 (Nox2) activation and oxidized LDL [108]. These data connect EVOO with attenuation of meal-induced inflammatory signaling, a setting in which postprandial lipemia and gut-derived LPS may converge.

Controlled fatty acid substitution studies help separate the contribution of the oleic acid-rich lipid environment from the whole EVOO matrix. Lowering the dietary palmitate-to-oleate ratio reduced LPS-stimulated peripheral blood mononuclear cells (PBMCs) production of inflammatory cytokines and decreased skeletal-muscle expression of redox-sensitive inflammatory genes in humans [118]. As noted above for neuroimmune outcomes, related palmitic acid-lowering/high-oleic acid interventions also reduced ex vivo inflammatory cytokine responses [117]. These studies support a role for dietary fatty acid composition in innate immune responsiveness, while EVOO-specific effects require consideration of the phenolic fraction and other minor compounds. Fish oil, docosahexaenoic acid (DHA), or conjugated linoleic acid (CLA) studies are best kept as external comparators showing that immune-cell function is sensitive to dietary lipid class. Preclinical and mechanistic studies linking EVOO or olive-derived compounds with barrier, inflammatory, metabolic, and gut–brain-related outcomes are summarized in Table 6.

To conclude, the immune evidence supports an anti-inflammatory role for EVOO, olive oil, olive-derived phenolics, and olive oil-rich dietary patterns, especially in metabolically or intestinally stressed settings, although comparative fat studies indicate that olive oil alone may not always reproduce the immune-regulatory effects of a broader Mediterranean-like lipid matrix. The strongest direct human evidence involves EVOO versus ROO and acute postprandial inflammatory responses, and the strongest mechanistic evidence comes from DSS colitis models and phenolic interventions. Future studies should determine whether systemic immune effects are driven by oleic acid, phenolic metabolites, reduced LPS exposure, direct effects on immune cells, or the combined EVOO matrix.

**Figure 2:**
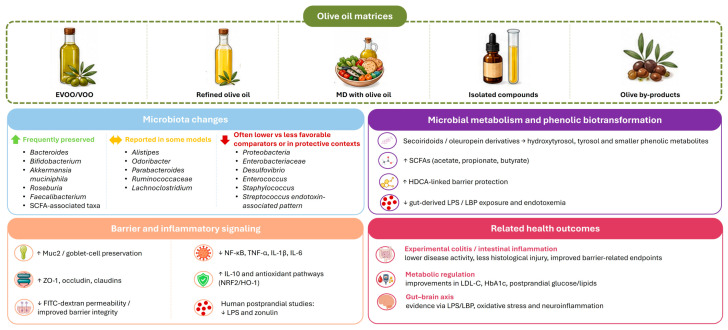
Summary of the main findings regarding olive oil and its effect on microbiota, metabolites, barrier and inflammatory signaling, and related health outcomes. Abbreviations: EVOO, extra-virgin olive oil; FITC-dextran, fluorescein isothiocyanate–dextran; HbA1c, glycated hemoglobin; HDCA, hyodeoxycholic acid; HO-1, heme oxygenase-1; IL, interleukin; LBP, lipopolysaccharide-binding protein; LDL-C, low-density lipoprotein cholesterol; LPS, lipopolysaccharide; MD, Mediterranean diet; Muc2, mucin 2; NF-κB, nuclear factor kappa B; NRF2, nuclear factor erythroid 2-related factor 2; SCFA, short-chain fatty acid; SCFAs, short-chain fatty acids; TNF-α, tumor necrosis factor alpha; VOO, virgin olive oil; ZO-1, zonula occludens-1. ↑ and ↓ means, respectively, increase or decrease associated with olive oil intervention or intake.

Figure 2 summarizes the main findings regarding olive oil, gut microbiota, microbial metabolites, barrier and inflammatory signaling, and related health outcomes.

## 9. Conclusions and Future Perspectives

Current evidence supports olive oil, particularly VOO and EVOO, as a microbiota-active dietary fat whose biological effects extend beyond its oleic acid-rich lipid matrix and involve complex interactions between minor bioactive compounds, gut microbial metabolism, and host physiology. Rather than promoting a unique microbial signature, olive oil appears to modulate microbial ecology and function in a context-dependent manner, influenced by oil quality, phenolic composition, dietary background, and host condition. Collectively, the available evidence suggests that preserving the minor bioactive fraction of virgin olive oils may contribute to microbiota-associated effects beyond those attributable to fatty acid composition alone, although the relative contribution of the lipid matrix and individual minor compounds remains incompletely understood.

Despite the rapid expansion of this field, several important limitations prevent definitive conclusions. The available evidence is dominated by preclinical studies, whereas well-designed human intervention trials remain scarce. In addition, substantial heterogeneity exists regarding olive oil characterization, intervention duration, study populations, dietary comparators, and microbiota analytical methods, limiting direct comparisons across studies. The limited number of investigations directly comparing EVOO, VOO, and ROOs also hinders the identification of the specific role of olive oil quality and minor bioactive constituents. Furthermore, many studies rely primarily on taxonomic descriptions of the gut microbiota, while functional analyses integrating microbial metabolism, microbial-derived metabolites, and host responses remain comparatively limited. Consequently, the biological significance of many reported taxonomic changes has yet to be established.

Future research should move beyond descriptive microbiota profiling toward integrated mechanistic and clinically relevant approaches capable of establishing causal relationships between olive oil consumption, microbial metabolism, and host physiology. Well-designed randomized controlled trials directly comparing chemically well-characterized EVOO, VOO, ROO, and appropriate oleic acid-rich control oils under standardized dietary conditions are required to disentangle the contribution of fatty acids and minor bioactive compounds. These studies should integrate gut microbiota composition with microbial and phenolic metabolomics, bile acid profiling, intestinal permeability assessment, immune and inflammatory biomarkers, and clinically meaningful outcomes. In addition, greater attention should be paid to interindividual variability in microbial metabolism and phenolic biotransformation, as these factors are likely to influence individual responsiveness to olive oil interventions. Ultimately, combining comprehensive olive oil characterization with multi-omics approaches and precision nutrition strategies will be essential to determine whether modulation of the gut microbiota represents a reproducible mechanism through which olive oil contributes to intestinal and systemic health.

## Figures and Tables

**Table 1:** Physicochemical characteristics and major chemical composition of virgin olive oil (VOO) and extra virgin olive oil (EVOO).

Parameter/Component	VOO	EVOO	Notes
Physicochemical quality parameters			
Free acidity (% oleic acid)	≤2.0	≤0.8	Classification criterion
Peroxide value (meq O_2_/kg)	≤20	≤20	Primary oxidation indicator
K232	≤2.60	≤2.50	Primary oxidation products
K270	≤0.25	≤0.22	Secondary oxidation products
ΔK	≤0.01	≤0.01	Oil authenticity/quality
Major fatty acids (% total fatty acids)			
Oleic acid (C18:1 *n*-9)	55–83	55–83	Main MUFA
Palmitic acid (C16:0)	7.5–20	7.5–20	Saturated fatty acid
Stearic acid (C18:0)	0.5–5	0.5–5	Saturated fatty acid
Linoleic acid (C18:2 *n*-6)	3.5–21	3.5–21	PUFA
α-Linolenic acid (C18:3 *n*-3)	<1	<1	Minor PUFA
Minor bioactive compounds			
Total phenolic compounds (mg/kg)	50–500	100–800	Strongly affected by cultivar and processing
Hydroxytyrosol	5–50	50–200	Includes free HT; total HT after hydrolysis may be substantially higher
Tyrosol (mg/kg)	5–30	10–50	Includes free tyrosol
Secoiridoids (oleuropein and ligstroside derivatives) (mg/kg)	50–350	100–600	Main phenolic fraction (oleacein, oleocanthal, and related aglycones)
Oleocanthal (mg/kg)	5–200	10–500	High variability among cultivars and harvests
Tocopherols (mainly α-tocopherol) (mg/kg)	100–300	100–300	Vitamin E activity
Phytosterols (mg/kg)	1000–2200	1000–2200	Mainly β-sitosterol
Squalene (mg/kg)	2000–7000	2000–7000	Major triterpene hydrocarbon
Pigments (chlorophylls, carotenoids) (mg/kg)	1–40	5–60	Usually higher in early-harvest EVOO

Data compiled from the literature [16,17,23,29,30,31]. Values represent typical ranges and may vary according to cultivar, geographical origin, agronomic conditions, fruit ripeness, extraction technology, and storage conditions. Abbreviations: ΔK, difference in specific extinction coefficients; EVOO, extra virgin olive oil; HT, hydroxytyrosol; K232, specific extinction coefficient at 232 nm; K270, specific extinction coefficient at 270 nm; meq O_2_/kg, milliequivalents of active oxygen per kilogram of oil; mg/kg, milligrams per kilogram; MUFA, monounsaturated fatty acid; PUFA, polyunsaturated fatty acid; VOO, virgin olive oil.

**Table 2:** Comparative studies evaluating the effects of different olive oil categories on gut microbiota and related outcomes.

Model	Comparison	Intervention	Main Microbiota Findings	Ref.
Male ICR/CD-1 mice	EVOO vs. ROO	HFD (35% kcal), 12 weeks	Distinct fecal microbial profiles despite similar fatty acid composition.	[49]
Male Swiss Webster ICR mice	EVOO vs. ROO	HFD (35% kcal), 12 weeks	ROO showed higher *Desulfovibrionaceae*, *Spiroplasmataceae*, and *Helicobacteraceae*; several taxa correlated with total cholesterol	[50]
Male Swiss Webster ICR mice	EVOO vs. ROO	HFD (35% kcal), 6 weeks	EVOO showed lower *Proteobacteria* and differences in several taxa; microbial changes were associated with glucose, leptin, and lipid parameters	[51]
Humans, older adults	VOO/EVOO vs. common OO	Observational	Higher α-diversity, *Bacteroides*, *Phascolarctobacterium*, and *Acidaminococcus*, and better cognitive trajectories	[33]

Abbreviations: EVOO, extra virgin olive oil; VOO, virgin olive oil; ROO, refined olive oil; HFD, high-fat diet.

**Table 3:** Preclinical whole-oil and dietary fat comparator studies evaluating the effects of olive oil, EVOO, and other dietary fats on gut microbiota, microbial metabolites, and host metabolic or inflammatory outcomes.

Model	Intervention	Main Observations	Interpretation	Ref.
Male Swiss Webster ICR/CD-1 mice	HFD with 20% EVOO/ROO/butter; 3 months	EVOO, ROO, and butter modified fecal microbiota differently; EVOO diverged more clearly from butter	Early evidence that oil refinement and minor compounds influence microbial response	[49]
Male Swiss Webster ICR mice	HFD with EVOO/butter (35% kcal); 12 weeks	Butter showed higher Desulfovibrionaceae/*Desulfovibrio* and worse metabolic phenotype than EVOO	Supports a more favorable EVOO response than saturated fat-rich butter	[57]
Male Swiss Webster ICR/CD-1 mice	HFD with 20% EVOO/ROO/butter; 12 weeks	ROO associated with higher Desulfovibrionaceae, Spiroplasmataceae, and Helicobacteraceae	Supports distinction between EVOO and ROO beyond fatty acid profile	[50]
Male Swiss Webster ICR mice	HFD with 35% energy from EVOO/ROO/butter; 12 weeks; microbiota at 6 weeks	EVOO showed lower Proteobacteria than butter and ROO; EVOO–ROO differences involved several taxa	Direct oil-category comparison supporting the contribution of EVOO minor compounds	[51]
Male C57BL/6J mice	HFD with 45% energy from EVOO/palm oil/safflower oil, flaxseed/fish oil; 16 weeks	Olive oil increased Bacteroidaceae/*Bacteroides* compared with palm oil and was associated with lower adiposity, but not higher diversity	Olive oil differs from saturated fat-rich comparators, but diversity is not a universal marker	[58]
Male C57BL/6J mice	HFD with 45% energy from olive oil/lard oil/soybean oil; 12 weeks	Olive oil showed milder oxidative and lipid disruption than lard, but still differed from a normal diet	Olive oil attenuates some HFD effects but does not normalize the phenotype	[59]
Male C57BL/6 WT and PPARα-null mice	7% or 21% EVOO/soybean oil/coconut oil; 3 months	EVOO increased favorable taxa and insulin-related outcomes; excessive oil reduced alpha diversity	Dose and comparator oil strongly condition the response	[60]
C57BL/6J mice with HFD-induced obesity/intestine stress	Lard-based HFD partially replaced with olive, perilla, or safflower oil (60% kcal fat); 16 weeks	Olive oil reduced body-weight gain, fat mass, leptin, Enterobacteriaceae, and serum endotoxin; perilla showed stronger bifidogenic effects	Olive oil improved selected HFD outcomes but was not the strongest comparator	[61]
Male C57BL/6J mice	HFD with 35% energy from EVOO/lard/flaxseed oil; 10 weeks	EVOO increased alpha diversity vs. lard and altered *Bacteroides*, *Allobaculum*, *Lachnospiraceae*, and *Mucispirillum*; reduced hepatic LBP	Links EVOO microbiota changes with mucosal immune regulation and reduced microbial-product exposure	[62]
Female CD1 mice	HFD with 60% energy from EVOO/coconut/sunflower oils; 16 weeks	EVOO reduced Proteobacteria and potentially pro-inflammatory genera, and better preserved *Akkermansia muciniphila*	EVOO may improve inflammation-related microbial features despite reduced diversity	[63]
Female BALB/c mice	HFD with 45% kcal from lard/olive/soybean oils; 27 weeks	Fat source-dependent microbiota shifts were more strongly linked to fecal long-chain fatty acids than IgA coating	Luminal lipid environment may contribute to microbiota restructuring	[65]
*Muc2*^−/−^ mice	Mediterranean-like fat blend, olive oil, corn oil, or milk fat; 12 weeks after weaning	Mediterranean-like blend associated with *Lactobacillus animalis*, Muribaculaceae, and *Alistipes*; individual-fat diets associated with Enterobacteriaceae and *Bacteroides massiliensis*	Olive oil alone did not reproduce the microbial profile of a broader Mediterranean-like fat blend	[66]
Rat model of diet-induced metabolic syndrome	10% EVOO in diet + 10% fructose water; 12 weeks	EVOO modulated taxa, including Bifidobacteriaceae/*Bifidobacterium*, Eubacteriaceae, Anaeroplasmataceae, Coriobacteriaceae, *Enterococcus*, and *Bilophila*	Supports host metabolic status as a modifier of EVOO–microbiota response	[68]
Female NOD mice	2.5 mL/kg/day EVOO; 14 weeks	Increased Bacteroidetes/Firmicutes ratio and SCFA-producing taxa such as *Lachnoclostridium* and *Ruminococcaceae_UCG-005*	Links EVOO with glucose-related outcomes and SCFA-associated microbiota in the autoimmune diabetes model	[69]

Abbreviations: EVOO, extra virgin olive oil; HFD, high-fat diet; IgA, immunoglobulin A; LBP, lipopolysaccharide-binding protein; NOD, non-obese diabetic; PPARα, peroxisome proliferator-activated receptor alpha; ROO, refined olive oil; SCFA, short-chain fatty acid; WT, wild-type.

**Table 4:** Studies evaluating olive-derived phenolics, olive by-products, and microbial biotransformation in simulated digestion, fermentation, ex vivo, preclinical, and human settings.

Model/System	Intervention	Main Observations	Interpretation	Ref.
Simulated digestion and fecal fermentation with porcine fecal inoculum	OLE-rich olive leaf extracts, EVOO as reference; in vitro digestion + 20 h fermentation	OLE decreased; HT and other low-molecular-weight metabolites increased; Coriobacteriaceae and *Collinsella* were affected	Direct support for microbial transformation of olive phenolics and EVOO secoiridoids	[83]
Ex vivo fecal fermentation with mouse feces	10 mg oleocanthal/250 mg feces; up to 4.5 h	Oleocanthal transformed into oleoglycine, tyrosol acetate, and tyrosol	Supports microbial transformation of an EVOO secoiridoid	[84]
Simulated gastrointestinal–colon model	HT and tyrosol; up to 24 h colonic incubation	HT generated a broader metabolite profile than tyrosol; both remained detectable after 24 h	Shows lower-gut availability and microbial transformation of simple olive phenols	[85]
Simulated digestion and fecal fermentation	HT digestion + fermentation sampled up to 48 h	HT generated phenolic- and indole-related metabolites and increased total SCFA	Supports HT as a microbiota-accessible phenolic	[86]
Mice and fecal microbiota assays	500 mg/kg tyrosol-equivalent; GI distribution up to 360 min; fermentation up to 72 h	Esters reached the cecum/colon and were hydrolyzed by fecal microbiota and *Lactobacillus* strains; free tyrosol was rapidly absorbed	Esterification may increase lower-gut delivery of olive phenols	[87]
In vitro digestion and fecal microbiota hydrolysis	HT-SCFA and tyrosol-SCFA acyl esters; simulated digestion up to 120 min; fermentation up to 72 h	Esters released olive phenolics and SCFAs in a structure-dependent manner	Mechanistic evidence for microbial enzymatic release of phenolic derivatives	[88]
SHIME model	Olive pâté; in vitro	Increased Lactobacillaceae and Bifidobacteriaceae; transformation of HT-related compounds	Olive by-products can act as phenolic-rich microbiota-active matrices	[89]
Ex vivo human colonic fermentation	Debittered olive pâté enriched with *Lactiplantibacillus plantarum*; 24 h colonic fermentation after simulated digestion	Modulated human colonic microbiota; prebiotic, eubiotic, and bifidogenic activity	By-product/probiotic matrix; not directly equivalent to whole EVOO	[90]
Mouse-derived *Enterococcus* isolates and in vitro assays	EVOO/ROO/butter dietary exposure; OLE/HT in vitro; mouse diet 12 weeks; in vitro 24 h	Dietary fat source influenced phenotypic traits of *Enterococcus* isolates	Suggests olive oil effects may occur at the strain or within-genus functional level	[97]
Humans with mild hypercholesterolemia	Olive pomace-enriched biscuits; 8 weeks	Limited diversity changes; trends toward increased *Bifidobacterium* and phenolic metabolites	Human by-product evidence for phenolic metabolism with limited community restructuring	[92]

Abbreviations: EVOO, extra virgin olive oil; GI, gastrointestinal; HT, hydroxytyrosol; OLE, oleuropein; ROO, refined olive oil; SCFA, short-chain fatty acid; SHIME, simulator of the human intestinal microbial ecosystem.

**Table 5:** Human studies evaluating olive oil, olive oil-rich dietary patterns, olive by-products, or related lipid exposures in relation to gut microbiota, microbial metabolites, postprandial metabolism, inflammation, and neurological outcomes.

Population/Model	Intervention	Main Observations	Interpretation	Ref.
Patients undergoing coronary angiography	25 mL/d polyphenol-rich EVOO/ROO; 6 weeks	EVOO: ↓ Total cholesterol, LDL-c, and CRP ↑ LPS-stimulated IL-10 compared with ROO	Direct human evidence that oil quality and phenolic content influence metabolic and inflammatory markers	[106]
Patients with metabolic syndrome	Three single-breakfast interventions with 40 mL VOO of high, intermediate, or low phenolic content; 1-week washout between breakfasts; 4 h postprandial follow-up.	↓ Postprandial LPS and TLR4/NF-κB-related inflammatory activation	Supports phenol-rich VOO in reducing postprandial endotoxemia and inflammatory signaling	[107]
Patients with impaired fasting glucose	Two isoenergetic lunches, with or without 10 g EVOO; postprandial assessment.	↓ Postprandial LPS, Apo-B48, Nox2 activation and oxidized LDL	Links EVOO with postprandial endotoxemia, lipoprotein handling, and oxidative stress	[108]
Patients with impaired fasting glucose	Mediterranean-type meal ±10 g EVOO in healthy subjects and IFG patients; washout 30 days or ≥7 days; 2 h follow-up; chocolate ± EVOO test in IFG patients.	↓ LPS and zonulin improved glucose, insulin, and GLP-1 responses	Suggests interaction between EVOO, gut permeability-related markers, and postprandial metabolism	[109]
Older patients with HIV	50 mL/day EVOO, 12 weeks	No major global microbiota shift; ↑ alpha diversity in men; ↓ Dethiosulfovibrionaceae, *Mogibacterium*, and *Coprococcus*	Direct EVOO evidence in a specific clinical population; limited by sample and context	[54]
Adults in pre–post intervention	30 mL/day polyphenol-rich EVOO, 100 days	↑ Fecal Bacteroidota and shifts in salivary microbiota; ↓ Reduced HbA1c, LDL-c and salivary IL-1β	Suggests microbial and systemic effects, but the uncontrolled design limits causal inference	[55]
CKD patients undergoing hemodialysis	40 mL/day EVOO, 14 days	↑ Fecal total SCFA and butyrate contribution No taxonomic microbiota profiling	Evidence for microbial metabolite modulation rather than direct microbiota composition	[56]
Normal-weight and overweight/obese adults	40 g/day EVOO in an MD, 3 months	↑ Fecal lactic acid bacteria Improved oxidative/inflammatory markers	Pattern-based intervention; EVOO contribution cannot be fully isolated	[105]
Obese men with coronary heart disease	An MD rich in olive oil vs. a low-fat/high-complex-carbohydrate diet; 1 year	MD reduced *Prevotella* and increased *Roseburia* and *Oscillospira*; improved insulin sensitivity	Supports olive oil-rich Mediterranean diet effects on microbiota and metabolism	[102]
Men with coronary heart disease and different metabolic statuses	MD vs. low-fat diet; 2 years	Dysbiosis improved mainly in obese subjects with severe metabolic dysfunction; MD increased *Roseburia*, *Ruminococcus*, *P. distasonis*, and *F. prausnitzii*	Baseline metabolic status modifies microbiota responsiveness	[74]
Adults with different habitual diets	Mediterranean dietary adherence; cross-sectional; 7-day dietary record	Higher Mediterranean adherence and plant-food intake are associated with higher fecal SCFA and taxa such as *Prevotella*, *Lachnospira*, and *Roseburia*	Supports the broader Mediterranean pattern, not olive oil alone	[13]
Adults without declared pathology	MD score cross-sectional FFQ	Higher adherence associated with higher Bacteroidetes, Prevotellaceae, *Prevotella*, and fecal propionate/butyrate	Pattern-based evidence linking the Mediterranean diet with SCFA-related profiles	[103]
PREDIMED-Plus participants with overweight/obesity and metabolic syndrome	Habitual VOO/EVOO vs. common olive oil intake; Baseline microbiota; 2-year cognitive follow-up	VOO/EVOO is associated with higher alpha diversity and more favorable cognitive trajectories; common olive oil showed less favorable associations	Human evidence supporting the separation of olive oil categories; observational	[33]
Pediatric epilepsy	Olive oil-rich Mediterranean ketogenic diet (>50% energy from olive oil), 3 months	Genus-level microbial shifts and SCFA-related changes without major alpha/beta diversity changes	Neurological dietary-pattern context; effects cannot be separated from ketosis and carbohydrate restriction	[75]
Mild hypercholesterolemia	Olive pomace-enriched biscuits 8 weeks	No major alpha/beta diversity changes; trends toward increased *Bifidobacterium* and phenolic metabolites	Olive by-products may affect phenolic metabolism even without broad community restructuring	[92]

Abbreviations: Apo-B48, apolipoprotein B48; CKD, chronic kidney disease; CRP, C-reactive protein; EVOO, extra virgin olive oil; FFQ, food-frequency questionnaire; GLP-1, glucagon-like peptide-1; HbA1c, glycated hemoglobin; HIV, human immunodeficiency virus; IFG, impaired fasting glucose; IL-1β, interleukin-1 beta; IL-10, interleukin-10; LDL-C, low-density lipoprotein cholesterol; LPS, lipopolysaccharide; MD, Mediterranean diet; NF-κB, nuclear factor kappa B; Nox2, NADPH oxidase 2; PREDIMED-Plus, PREvención con DIeta MEDiterránea-Plus; ROO, refined olive oil; SCFA, short-chain fatty acid; TLR4, Toll-like receptor 4; VOO, virgin olive oil. ↑ and ↓ means, respectively, increase or decrease associated with olive oil intervention or intake.

**Table 6:** Preclinical and mechanistic studies linking EVOO, olive oil, or olive-derived compounds with intestinal barrier function, experimental colitis, metabolic dysfunction, immune regulation, and gut–brain-related outcomes.

Model	Intervention	Main Observations	Interpretation	Ref.
DSS-induced colitis in mice	EVOO daily by gavage, DSS 5%; 10 days	↓ Body-weight loss, rectal bleeding, histological damage, FITC-dextran permeability, and inflammatory gene expression	Strongest whole EVOO evidence for barrier protection in experimental colitis	[110]
DSS-induced colitis in mice	HT 40 mg/kg/d by gavage for 14 days; 3% DSS for 9 days; sacrifice on day 15.	↓ DAI, colon shortening, histological damage, and apoptosis; ↑ Improved antioxidant defenses; Inhibited NLRP3; Restored microbiota diversity and SCFA.	Supports HT effects on inflammation, microbiota, and SCFA recovery	[98]
DSS-induced colitis in mice	HT 10 or 50 mg/kg; 3% DSS in final 7 days; 14 days	↓ MPO and cytokines; ↑ IL-10, activated Nrf2/HO-1, Muc2 and tight-junction proteins ↓ TLR4/p65 NF-κB activation	Convergent antioxidant, anti-inflammatory, and barrier-related mechanisms	[99]
DSS-induced colitis in mice	Tyrosol 20 mg/kg/d; *Lactobacillus plantarum* SC-5 1 × 10^10^ CFU/kg/d; 14 days	↓ Disease activity, oxidative stress, and cytokines; Preserved ZO-1, occludin and claudin-3; FMT transferred part of the protection	Supports microbiota-dependent protection, but the effect cannot be attributed to tyrosol alone	[101]
DSS-induced colitis in mice	OLE 20 mg/kg/d during 7 days of 3% DSS exposure; FMT for 1 week using feces from OLE-treated donors; HDCA 50 mg/kg in the validation experiment.	↓ DAI, colon shortening, cytokines, MPO, and MDA; Restored ZO-1 and claudin-3; FMT/HDCA supported microbiota–bile acid–barrier mechanism	Strong mechanistic evidence linking olive phenolics, bile acids, microbiota, and barrier protection	[100]
*Muc2*^−/−^ spontaneous colitis model	Mediterranean-like fat blend vs. olive oil, corn oil, or milk fat; 12 weeks	Mediterranean-like blend reduced disease activity, histological damage, ulceration, and crypt abscesses more consistently than olive oil alone	Protection depended on a balanced fatty acid pattern, not olive oil alone	[66]
Diquat-challenged piglets and IPEC-J2 cells	HT 500 mg/kg diet; 28 days; diquat on day 21	Preserved intestinal morphology and tight-junction proteins; ↓ Permeability-related damage; ↑ PI3K/Akt-Nrf2 and mitophagy	Evidence for epithelial redox protection more than microbiota-mediated modulation	[111]
EAE in Dark Agouti rats	EVOO 10% or oleic acid 4% of caloric intake, or HT 0.5 mg/kg/d; 51 days	↓ LPS/LBP in blood, brain, and spinal cord, and lowered oxidative/inflammatory markers	Evidence for gut-derived inflammatory signaling, not direct microbiota remodeling	[71]
EAE mouse model and Caco-2 cells	Oleacein 10 mg/kg/d (i.p); 24 days	↓ FITC-dextran permeability, serum iFABP and sCD14; Preserved mucin staining; ↓ TNFα-induced barrier dysfunction	Supports barrier-protective potential of olive secoiridoids in neuroinflammatory context	[112]
AlCl_3_-induced mild cognitive impairment in rats	EVOO 3.0 mL/kg/d; 49 days	Improved cognition; ↓ Brain inflammatory/oxidative markers; ↑ *Alistipes*, *Odoribacter*, and *Parabacteroides*	Suggests gut–brain relevance, but microbiota causality remains unresolved	[73]
Scopolamine-induced AD model in male mice	10-week HFDs supplemented with 47% EVOO, ROO, or RPO, or 32% ROO + 15% fish oil; scopolamine 1 mg/kg i.p. during final week	All HFD groups attenuated scopolamine-induced increases in Iba-1, COX-2, and TNF-α; EVOO-HFD showed the lowest GFAP signal and more favorable astroglial morphology, followed by ω3-LCPUFA	Supports lipid-quality and oil-matrix effects on hippocampal neuroinflammation, but not direct microbiota-mediated gut–brain evidence	[114]
Chronic mild stress in rats	Diet with 2% EVOO + 2% soybean oil vs. fish oil; 14 weeks	EVOO modified stress-associated microbiota, including *Akkermansia*, *Romboutsia*, and *Ruminococcaceae_UCG_003*; fish oil showed clearer behavioral effects	EVOO modulates gut–brain readouts but is not the strongest lipid comparator	[64]
Chronic unpredictable mild stress model in rats	Olive oil-containing diet vs. fish oil 4% fat by weight; 14 weeks	Olive oil altered microbiota and selected neurotransmitter/inflammatory markers; Fish oil produced stronger behavioral and barrier effects	Supports lipid-quality effects in the gut–brain axis with comparator-dependent efficacy	[72]
Traumatic-stress model in aged mice	HT 100 mg/kg/d; 30 or 50 days	↓ Anxiety-like responses and neuroinflammation; Preserved stress-sensitive microbial families	Supports possible microbiota–gut–brain contribution of HT in stress resilience	[96]
HFD-induced obesity in mice	Tyrosol 0.2% *w*/*w*; 16 weeks	Partially normalized dysbiosis, increased *Verrucomicrobia*, and improved obesity-related metabolic and thermogenic markers	Isolated phenolic evidence for metabolic and microbiota modulation	[93]
PM2.5-induced metabolic dysfunction in mice	HT 50 mg/kg/d; 4 weeks	Restored microbial richness and counteracted metabolic dysfunction	Supports microbiota-associated antioxidant/metabolic protection by isolated HT	[94]

Abbreviations: AD, Alzheimer’s disease; CFU, colony-forming units; COX-2, cyclooxygenase-2; DAI, disease activity index; DSS, dextran sodium sulfate; EAE, experimental autoimmune encephalomyelitis; EVOO, extra virgin olive oil; FITC-dextran, fluorescein isothiocyanate–dextran; FMT, fecal microbiota transplantation; GFAP, glial fibrillary acidic protein; HDCA, hyodeoxycholic acid; HFD, high-fat diet; HO-1, heme oxygenase-1; HT, hydroxytyrosol; Iba-1, ionized calcium-binding adaptor molecule 1; iFABP, intestinal fatty acid-binding protein; IL-10, interleukin-10; i.p., intraperitoneal; IPEC-J2, intestinal porcine epithelial cell line J2; LBP, lipopolysaccharide-binding protein; LPS, lipopolysaccharide; MDA, malondialdehyde; MPO, myeloperoxidase; *Muc2*^−/−^, mucin-2-deficient; NF-κB, nuclear factor kappa B; NLRP3, NOD-, LRR- and pyrin domain-containing protein 3; Nrf2, nuclear factor erythroid 2-related factor 2; PI3K, phosphoinositide 3-kinase; ROO, refined olive oil; RPO, refined palm oil; SCFA, short-chain fatty acid; sCD14, soluble CD14; TLR4, Toll-like receptor 4; TNFα, tumor necrosis factor alpha; ω3-LCPUFA, omega-3 long-chain polyunsaturated fatty acids; ZO-1, zonula occludens-1. ↑ and ↓ means, respectively, increase or decrease associated with olive oil intervention or intake.

## Data Availability

Data sharing is not applicable to this article as no datasets were generated or analyzed during the current study.

## Abbreviations

The following abbreviations are used in this manuscript:

CKD	Chronic kidney disease
CLA	Conjugated linoleic acid
COX-2	Cyclooxygenase-2
CORDIOPREV	Coronary Diet Intervention With Olive Oil and Cardiovascular Prevention
CRP	C-reactive protein
DAI	Disease activity index
DGGE	Denaturing gradient gel electrophoresis
DHA	Docosahexaenoic acid
DOPAC	3,4-dihydroxyphenylacetic acid
DSS	Dextran sodium sulfate
EAE	Experimental autoimmune encephalomyelitis
EFSA	European Food Safety Authority
EVOO	Extra-virgin olive oil
F/B ratio	Firmicutes-to-bacteroidetes ratio
FITC-dextran	Fluorescein isothiocyanate–dextran
FMT	Fecal microbiota transplantation
GDNF	Glial cell line-derived neurotrophic factor
GLP-1	Glucagon-like peptide-1
HDCA	Hyodeoxycholic acid
HFD	High-fat diet
HIV	Human immunodeficiency virus
HO-1	Heme oxygenase-1
HT	Hydroxytyrosol
IBD	Inflammatory bowel diseases
IgA	Immunoglobulin A
iFABP	Intestinal fatty acid-binding protein
IL	Interleukin
iNOS	Inducible nitric oxide synthase
LBP	Lipopolysaccharide-binding protein
LDL-c	Low-density lipoprotein cholesterol
LPS	Lipopolysaccharide
MAPK	Mitogen-activated protein kinase
MD	Mediterranean diet
MDA	Malondialdehyde
MPO	Myeloperoxidase
MUFA	Monounsaturated fatty acid
NF-κB	Nuclear factor kappa B
NOD	Non-obese diabetic
Nox2	NADPH oxidase 2
Nrf2	Nuclear factor erythroid 2-related factor 2
PBMCs	Peripheral blood mononuclear cells
PPARα	Peroxisome proliferator-activated receptor alpha
PREDIMED	PREvención con DIeta MEDiterránea
PUFA	Polyunsaturated fatty acid
ROO	Refined olive oil
SCFA	Short-chain fatty acid
SHIME	Simulator of the human intestinal microbial ecosystem
TLR4	Toll-like receptor 4
TNF-α	Tumor necrosis factor alpha
VOO	Virgin olive oil
ZO-1	Zonula occludens-1
